# Correlations of Behavioral Deficits with Brain Pathology Assessed through Longitudinal MRI and Histopathology in the R6/1 Mouse Model of Huntington’s Disease

**DOI:** 10.1371/journal.pone.0084726

**Published:** 2013-12-19

**Authors:** Ivan Rattray, Edward J. Smith, William R. Crum, Thomas A. Walker, Richard Gale, Gillian P. Bates, Michel Modo

**Affiliations:** 1 King’s College London, Institute of Psychiatry, Department of Neuroscience, London, United Kingdom; 2 King’s College London, Department of Medical and Molecular Genetics, London, United Kingdom; 3 King’s College London, Department of Neuroimaging, Institute of Psychiatry, London, United Kingdom; 4 University of Pittsburgh, Department of Radiology, McGowan Institute for Regenerative Medicine, Pittsburgh, Pennsylvania, United States of America; University of Florida, United States of America

## Abstract

Huntington’s disease (HD) is caused by the expansion of a CAG repeat in the huntingtin (*HTT*) gene. The R6 mouse models of HD express a mutant version of exon 1 *HTT* and typically develop motor and cognitive impairments, a widespread huntingtin (HTT) aggregate pathology and brain atrophy. Unlike the more commonly used R6/2 mouse line, R6/1 mice have fewer CAG repeats and, subsequently, a less rapid pathological decline. Compared to the R6/2 line, fewer descriptions of the progressive pathologies exhibited by R6/1 mice exist. The association between the molecular and cellular neuropathology with brain atrophy, and with the development of behavioral phenotypes remains poorly understood in many models of HD. In attempt to link these factors in the R6/1 mouse line, we have performed detailed assessments of behavior and of regional brain abnormalities determined through longitudinal, *in vivo* magnetic resonance imaging (MRI), as well as an end-stage, *ex vivo* MRI study and histological assessment. We found progressive decline in both motor and non-motor related behavioral tasks in R6/1 mice, first evident at 11 weeks of age. Regional brain volumes were generally unaffected at 9 weeks, but by 17 weeks there was significant grey matter atrophy. This age-related brain volume loss was validated using a more precise, semi-automated Tensor Based morphometry assessment. As well as these clear progressive phenotypes, mutant HTT (mHTT) protein, the hallmark of HD molecular pathology, was widely distributed throughout the R6/1 brain and was accompanied by neuronal loss. Despite these seemingly concomitant, robust pathological phenotypes, there appeared to be little correlation between the three main outcome measures: behavioral performance, MRI-detected brain atrophy and histopathology. In conclusion, R6/1 mice exhibit many features of HD, but the underlying mechanisms driving these clear behavioral disturbances and the brain volume loss, still remain unclear.

## Introduction

Huntington’s disease (HD), a progressive, autosomal dominant neurological disorder, is caused by a CAG/polyglutamine repeat expansion [1]. This unstable elongation leads to the eventual aggregation of mutant huntingtin (mHTT), resulting in neurodegeneration and ultimately death. Even during the prodromal phase, subtle brain changes have been shown to be associated with disease burden [2,3,4]. Still, despite the genetic cause for HD being identified, the causal cascade linking the activity of mHTT with the development of clinical signs remains poorly understood. The varying nature of HD, as well as the scarcity of affected tissue during the early stages of disease, impede efforts to link molecular pathology to both brain atrophy and motor/behavioral dysfunction.

 To overcome such issues and improve our understanding of the causes of HD, a variety of rodent models have been developed. Transgenic mice include the R6 [5] and N171Q82 [6] lines that express N-terminal fragments of HTT, as well as the YAC128 [7] and BACHD [8] lines that express a mutant version of the full-length protein. The genetic basis of HD is more precisely recapitulated by the knock-in models in which an expanded CAG repeat has been inserted into mouse *Htt* and that develop a more slowly progressing phenotype [9,10,11]. There are several comparable late-stage phenotypes between R6/2 mice at 12-14 weeks and *Hdh*Q150 mice at 22 months of age [12,13,14,15,16]. Consequently, we defined the N-terminal mHTT fragments that are present in *Hdh*Q150 brain tissue and found that the smallest fragment is exon 1 protein [17]. We have subsequently shown that this is generated through the mis-splicing of exon 1, indicating that the R6 mice are a model for the aberrant splicing that occurs in HD [18]. This current study is part of a continuing detailed comparison of the progressive pathologies exhibited by the R6 and knock-in mouse models.

We have previously described the concomitant behavioral dysfunction and development of brain abnormalities detected through magnetic resonance imaging (MRI) exhibited by the R6/2 mouse line, expressing ~210 CAG repeats [19]. A similar, but lesser used mouse model, the R6/1 line, expresses fewer CAG repeats (typically ~110-120) and consequently exhibit a slower pathological progression and elongated life-span, compared to R6/2, up to 40 weeks [5]. These features potentially make it more suitable and sensitive to investigate early molecular changes and their relevance to behavioral impairments. R6/1 mice exhibit age-related progressive impairments at both motor and cognitive tests from as early as 2 months of age [20], and age related neuronal loss, brain atrophy and mHTT accumulation [21]. 

Recent efforts have been made to characterize disease burden in HD mouse models, including transgenic [20,21], BACHD [22], YAC128 [23,24,25,26], as well as the CAG knock-in mice of varying repeat lengths [27,28,29,30,31,32,33,34,35]. Such descriptions of pathological progression are essential for understanding how the construction of an HD mouse can lead to varying phenotypes. Nevertheless, establishing a causal relationship requires a longitudinal assessment of the emergence of behavioral dysfunction with concomitant measurement of brain atrophy by MRI, along with their correlation with cellular and molecular changes. We here present a correlative analysis of these concomitant changes in behavior and pathology in both female and male R6/1 mice. 

## Materials and Methods

### Ethics statement

All experimental procedures performed on mice were approved by the King’s College London Ethical Review Process Committee and carried out under Home Office License 70/6445.

### Animals

Hemizygous R6/1 mice were bred by backcrossing R6/1 males to (CBA x C57BL/6) F1 females (B6CBAF1/OlaHsd, Harlan Olac, Bicester, UK). Mice were genotyped and the CAG repeat size was measured, as described previously [15]. The CAG repeat was 123.19 (± 1.52, SD); the CAG repeat length did not differ between male (123.33 ± 0.866, SD) and female (123.0 ± 2.16) R6/1 mice. Littermates were divided into four groups: male wild type (WT, n= 11), male R6/1 (n=9), female WT (n=10) and female R6/1 (n=10), an equal ratio between male:female was aimed for. For exact numbers of subjects included at each stage of analysis see Table S1. All procedures reported here (behavioral, MRI and histological) were conducted on the same cohort of animals.

**Figure 1:**
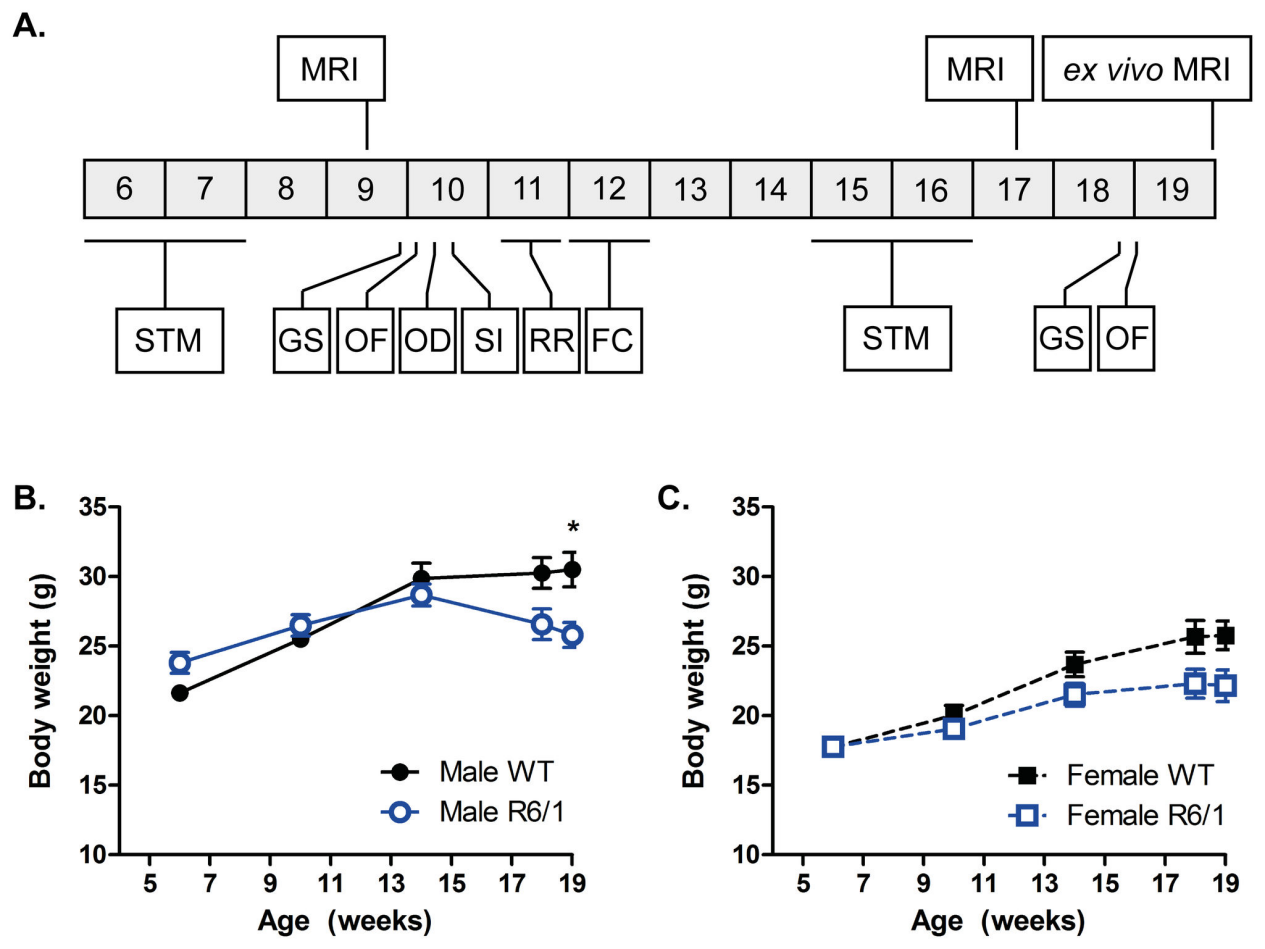
Experimental design and body weight measures. (A) Experimental design from 6 to 19 weeks of age. All mice were subjected to repeated assessments of behaviors, alongside *in* *vivo* MRI taken at 9 and 17 weeks of age. At 19 weeks of age all mice were culled and an *ex* *vivo* MRI scan was taken. STM = swimming T-maze; GS = grip strength; OF = locomotor activity in an open field, OD = odor discrimination, SI = social interaction, RR = rotarod, FC = cued and contextual fear conditioning. Body weight gain for both male (B) and female mice (C). Body weight analyzed using three-way ANOVA (Genotype X Gender X Age). Body weight: WT male n=11, R6/1 male n=9, WT female n=10, R6/1 female n=10-8. All data presented as means ± SEM; *p<0.05.

WT and R6/1 mice were housed under standard animal laboratory conditions. Room temperature was automatically maintained at 21°C ± 1°C. Animals were kept on a 12h light:dark cycle. Mice were group-housed dependent on gender, but genotypes were mixed within the cages. All mice had access to standard cage environmental enrichment (bedding and play tube) and maintained on a standard chow diet with tap water available *ad libitum*. Body weight was monitored at 6, 10, 14, 18 and 19 weeks of age. Body temperature was recorded up to 18 weeks of age through an infra-red temperature reader (ThermoScan Instant Thermometer, Braun) reproducibly positioned under the thorax. An overview of the experimental design is presented in Figure 1A. 

### Behavioral tests

Mice were exposed to repeated behavioral testing from 6 weeks of age (Figure 1A). Learning in a swimming T-maze was probed at both 6 and 15 weeks of age (~2 week procedure at both ages). Grip strength capacity and behaviors exhibited in an open field were tested twice, at 10 and 19 weeks. Olfactory capacity and discrimination of social novelty were investigated at 10 weeks only, and motor coordination and cognition were tested in the rotarod and fear conditioning paradigms at 11 and 12 weeks respectively.

### Motor-related tasks

#### Rotarod

Motor coordination was tested on an accelerating rotarod at 11 weeks of age, as described previously [19]. In brief, mice were individually placed on an accelerating, rotating beam (4-40 rpm) for a maximum of 300 sec. Latency to fall from the beam (sec) was recorded as a measure of motor performance. Mice were exposed to three trials per day for four consecutive days (day 1 behavior was considered habituation to the test and the data was subsequently discarded). The whole apparatus was thoroughly cleaned with 70% industrial methylated spirit (IMS) between trials.

#### Open field

General ambulation was probed in an open-field arena at 10 and 19 weeks of age. The test was conducted 2 h into the mouse’s dark cycle, under red-light conditions. Mice were habituated to the test room conditions for 2 h, then individually placed into square, plain white arenas (50 x 50 x 50 cm, Engineering & Design Plastics Ltd., Cambridge, UK) for 30 min, and behavior was recorded through a video camera positioned above the apparatus. Activity (distance moved, cm) was tracked and later analyzed using EthoVision 7XT software (Noldus, Netherlands). Arenas were thoroughly cleaned with 70% IMS between trials.

#### Grip strength

Grip strength capacity was assessed at both 10 and 19 weeks of age. The protocol allowed for the independent measurement of the strength of the forelimbs only, as well as fore- and hind limbs taken together. Mice were held by the base of the tail and gently swung to allow them to grip the wire-mesh grid attached to a grip strength monitor (Bioseb In Vivo Research Instruments). Mice were either allowed to grip with forelimbs, or fore- and hind limbs together, and then gently pulled away from the apparatus. Maximum tension (g) before the mouse released the grid was recorded. For both grip strength recordings (forelimb only, and fore- and hind limbs together), the mice were given three attempts and the average performance was taken for analysis. 

### Non-motor-related tasks

#### Swimming T-maze

Cue learning, and the reversal of cue learning was conducted in a swimming T-maze at 6 and 15 weeks of age, using an adapted protocol [24]. The maze consisted of a start corridor (122 cm length, 12 cm width, 35 cm depth made from black, opaque plastic) leading to a left and right choice arm (60 cm length, 12 cm width, 35 cm depth) forming a T-junction. The maze was filled with water made opaque through the addition of a whitening agent (Marvel dried skimmed milk, Premier Foods, UK). Water was replaced and the maze thoroughly cleaned every third day. A transparent plastic square escape platform was submerged 0.5 cm, invisible under the water surface. Water temperature was maintained at 21-25°C throughout the duration of the procedure. For “cue learning”, the escape platform was pseudo-randomly located (location order picked randomly out-of-a-hat), at either the left or right end of the top arm and was cued to the presence of an illuminated desktop lamp directly over the escape platform. Mice were individually placed in the starting position (at the base of the starting arm, opposite where it bisects the top arm) and allowed to swim to the T-junction of the two arms whereupon it made a decision to swim either toward the light (where it reached the escape platform and was removed from the maze), or away from the light (where it was forced to remain in the incorrect arm for 10 sec before being allowed to enter the correct side of the top arm and find the escape platform). Mice were towel dried between trials. Mice were exposed to 12 trails per day until they reached criterion (10 correct choices out of 12 successive trials (including rolling over from the previous day), 83.3% correct rate), whereby they began the second part of the trial, termed “cue reversal” learning. For cue reversal training, mice were exposed to the same conditions to those described for the first, cued learning part of the task, but the desktop lamp cue was now situated at the opposite arm to escape platform. Thus, mice were forced to re-learn the escape conditions. Cue reversal training was run until mice reached criterion (again, 83.3% correct rate), at which point the assessment was discontinued.

#### Fear conditioning

Cued and contextual fear conditioning was used to probe cognitive function in these mice at 12 weeks, using an adapted protocol [36]. Experiments were conducted using a standard fear conditioning system (TSE-Systems, Germany). The test was divided into three days, day 1 “training”, day 2 “cue recall and extinction” and day 3 “context recall”. 

#### Day 1 “training”

The operant chamber was scented using a solution of 79.5% water, 19.5% ethanol, 1% vanilla extract. Mice were individually placed in the arenas and exposed to the conditioning protocol; three pairings of a conditioned stimulus (30 sec auditory tone), and an unconditioned stimulus foot-shock (0.5mA, 1 sec duration). 

#### Day 2 “cue recall and extinction”

The operant chamber was thoroughly cleaned with 50% ethanol to remove all vanilla extract odor. Monochrome patterned wall inserts and a plain white floor insert were added to change the context of the operant chamber. Mice were individually placed in the chamber and subjected to 25 exposures of the conditioned stimulus (auditory tone), but no foot shock, in order to measure cued recall and extinction of conditioned fear. 

#### Day 3 “context recall”

The operant chamber was returned to the identical context as described for Day 1 (chamber inserts removed, and vanilla odor added) with mice being placed in the operant chamber for 5 min with no conditioned or unconditioned stimuli to determine contextual recall of conditioned fear. 

Throughout the duration of these tests, behavior was videotaped by a camera positioned above the apparatus. Mouse immobility (a complete absence of movement except breathing) was considered a measure of fear, scored by an investigator blinded to the experimental groupings. An inter-rater reliability of >95% confidence was achieved. For cued recall and extinction on Day 2, immobility over five summed CS exposures (giving a total of 5, 30 sec blocks) was used for analysis. For contextual recall on Day 3, immobility was expressed over the entire 5 min trial.

#### Odor discrimination

This test was conducted to probe olfactory function of mice at 10 and 19 weeks of age. Mice were habituated to plain white arenas (50 x 50 x 50 cm), for 10 min per day for two days prior to starting the test. On the test day, two samples of mouse litter were available for investigation over a total of 5 min, one sample being clean litter, the other being soiled litter from unfamiliar sex-matched mice. Samples of litter were held in up-turned eppendorf tubes with 0.5 cm of the tips removed to allow the animal to smell the contents. Behavior was video taped from above and subsequently analyzed. Time spent investigating the tubes (nose pokes directly at the aperture of the tubes) was quantified by an investigator blinded to both the samples and experimental groupings. Preference for the soiled litter was expressed as percentage of time investigating soiled sample out of total time investigating either sample. An inter-rater reliability of >95% was achieved. Arenas were thoroughly cleaned with 70% IMS between trials.

#### Social interaction

Social behaviors, as well as the ability to discriminate novelty, was tested at 10 weeks of age using an adapted protocol [37]. Test mice were habituated to plain white arenas (50 x 50 x 50 cm) for 1 h. Two corrals (up-turned pen holders, Staples, UK) were then positioned inside the arenas in reproducible positions, and the test mice were allowed to habituate to the corrals for a further 30 min. During “exposure 1”, a sex-matched, 8 week old C57Bl/6 mouse was positioned in one corral and the test mouse was allowed to investigate, but not physically interact with the mouse for 5 min. This now “familiar” mouse was removed, and following an inter-trial-interval of 30 min “exposure 2” was conducted, whereby the “familiar” mouse was replaced back into the same corral and a different, “novel” C57Bl/6 mouse was placed in the other corral, the test mouse was free to investigate either the familiar or novel mouse. Behavior was video-taped from above and subsequently analyzed. Time spent investigating the familiar and/or novel mice was measured by an investigator blinded to the experimental groups. An inter-rater reliability of >95% was achieved. Arenas and corrals were thoroughly cleaned with 70% IMS between trials.

### Magnetic Resonance Imaging

#### In vivo longitudinal MRI

WT and R6/1 mice were scanned twice *in vivo*, at 9 and 17 weeks of age. Mice were anaesthetized using 5% isoflurane along with a combination of medical air (0.7 l/min) and oxygen (0.3 l/min). Once fully anesthetized, mice were positioned and fixed into a plastic frame, where anesthetic was administered through a facemask. Mice were maintained under anesthesia, typically between 1-2% isoflurane for the duration of the scanning. Temperature was maintained through a homeostatic heating airflow system and breathing rate monitored through a respiration balloon positioned under the thorax (Small Animal Instruments, New York, USA). Post scanning, but prior to recovery, mice were administered 0.1ml saline i.p. to abate dehydration.

Images were acquired on a 7T horizontal bore Magnetic Resonance Imaging (MRI) system (Varian, Paolo Alto, California, USA), with a 100 Gauss gradient set insert and a 39 mm-bore (transmission and receiver) radiofrequency coil (Rapid, Germany). The scanner was controlled through VnmrJ software (Varian, Paolo Alto, California, USA). Correct positioning of the mouse within the RF coil was confirmed through a series of scouting images. A Multi-Echo-Multi-Slice (MEMS) scan was then acquired (*TR* = 2500 msec, *TE* = 10 msec, echo train = 8, averages = 4, matrix = 128 x 128, FOV = 20 x 20 mm, 30 coronal slices at 0.5 mm thickness, 21 min acquisition time). The typical signal-to-noise ratio (SNR) = 5.4, the typical white:grey matter ratio (WGR) = 1.25. Coronal slices were positioned based on a reproducible anatomical marker (the most visibly posterior part of the cerebellum). 

Post-acquisition, all eight echoes were summed into a single structural image set. As previously described, [19,38], these images were used to manually delineate neuroanatomical structures in JIM Ver. 5.0 (Xinapse Systems, Alwincle, UK). Regions-of-interest (ROIs) consisted of: whole brain, cortex, striatum, hippocampus, and corpus callosum. ROIs were delineated by two investigators blinded to the experimental groupings, and intra- and inter-rater reliability was consistently ≥ 95% confidence. Details of neuroanatomical inclusion criteria and delineation guidelines were identical to those described previously [19]. All information outside of the ROIs was subsequently masked out, the ROIs were then individually saved in NIFTI format. Volumetric data were calculated and processed semi-automatically using Python Ver.2.6 (Python Software Foundation). To measure changes in T2 relaxivity (reflective of tissue composition), maps of T2 signal intensity were obtained through a mono-exponential fit of the eight echoes. The ROIs were superimposed onto the maps of T2 signal intensity allowing for the generation of mean T2 relaxation times within each ROI. A small circular ROI was taken for cheek muscle tissue T2 relaxivity in order to act as an internal control measure. 

#### Ex vivo MRI

Post-mortem *ex vivo* MRI scans were taken at a higher-resolution compared to the *in vivo* scans and did not suffer from potential artefacts arising in live scanning (e.g. head movement). A more precise measurement of subtle changes in brain structures is therefore achievable. Following the final behavior test, at 19 weeks of age, mice were anaesthetized using terminal anesthesia (Euthatal, Marial, Harlow, UK), and then transcardially perfused with heparinized saline (50 units/ml), followed by Parafix (4% paraformaldehyde, Pioneer Research Chemical Ltd., Essex, UK). Whole heads were removed and submerged in Parafix and kept at 4°C until *ex vivo* imaging. The scanning set-up was identical to that used for *in vivo* imaging. Correct positioning of the mouse head within the RF coil was confirmed through a series of scouting images. An MEMS sequence was then acquired (*TR* = 3000 msec, TE = 10 msec, echo train = 8, averages = 22, matrix = 192 x 192, FOV = 19.2 x 19.2 mm, 35 coronal slices at 0.5 mm thickness, acquisition time for scan was ~3.5 hours). Typical SNR = 11.96, typical WGR = 1.54. Resulting images were subsequently converted to ANALYZE 7.5 for Tensor Based Morphometry (TBM).

#### Tensor Based Morphometry

An un-biased whole-brain comparison of WT and R6/1 at each imaging time-point was performed using an automated image processing pipeline [39]. All scans were first registered with 6 degrees of freedom (dof) (to remove differences in position and orientation using a rigid-body assumption) and 9 degrees of freedom (to optionally remove global differences in scale using a growth model based on uniform scaling) using a population based approach which has proven robust in rodent imaging applications [40]. Then each scan was non-rigidly registered to the WT mean at the same time-point using a high-dimensional fluid registration technique [41,42,43]. This technique models the coordinate mapping between scans as the flow of a viscous fluid and can successfully map large structural displacements as well as smaller localized changes in shape. This technique has previously been applied in other rodent models, e.g. structural remodeling in stroke [39]. The fluid registration results in a dense displacement field that maps each point in the original scan to the corresponding point on the reference mean. From this map, an estimate of apparent volume difference (the Jacobian determinant) between the scan and the WT mean at each voxel can be obtained. TBM analysis then applies voxel-wise non-parametric *t*-tests to these volume difference estimates to determine the location of statistically significant differences in brain tissue volume of R6/1 compared with WT. Significance levels are corrected for multiple comparisons across voxels using the False Discovery Rate. The analysis following 6 dof registration finds absolute volume differences (i.e. the fluid registration includes differences in brain size and the analysis finds absolute volume difference between regions), whereas 9 dof registration finds volume differences relative to whole brain volume, V (i.e. the fluid registration does not include differences in global brain volume and the analysis finds volume differences relative to whole brain volume). Collectively, these analyses allow for the comparison of WT versus R6/1 at each time point (three image sets in total, 9 and 17 weeks *in vivo* and 19 weeks *ex vivo*), as well as the age-related change within each genotype (from 9 to 17 weeks of age). A detailed methodological description of this approach can be found in [39].

### Histology

Upon completion of *ex vivo* imaging, brains were removed from the skulls, rinsed in phosphate buffered saline (PBS) and cryoprotected in 30% sucrose in PBS (+0.05% sodium azide) until sectioning. Coronal sections were taken serially at 50 μm thickness on a freezing microtome (HM430 Microm, Thermo Scientific) and stored at -20°C in tissue cryoprotective solution (+ 0.05% sodium azide) until staining. Histological processing and data collection was performed identically to that described previously in [19].

#### Immunohistochemistry

Sections were washed in PBS prior to incubation for 30 min in 3% H_2_O_2_ in PBS to quench endogenous peroxidase activity. Non-specific binding was blocked with a 1 h incubation in 10% normal serum with 0.3% Triton X-100 in PBS. Sections were then incubated overnight at 4°C in primary antibodies against NeuN (1:500, Millipore, Watford, UK) or S830 (1:2000), raised against exon 1 HTT with 53 glutamines [44], prior to incubation in appropriate biotinylated secondary antibody (Vector, Peterborough, UK) for 2 h at RT, followed by 1 h incubation in an avidin-biotinylated-peroxide complex (1:100, Vector, Northampton, UK). 3, 3'-diaminobenzidine (Sigma-Aldrich, Poole, UK) was used as the chromagen.

#### Cortical Thickness

Assessment of regional cortical atrophy was determined by thickness measurements of primary motor cortex (M1) and primary sensory cortex (S1) on NeuN-stained sections. In each region, 10 vertical lines were drawn covering all layers from the most dorsal horn of the corpus callosum to the pial surface. The mean length taken was for 3 consecutive sections caudally from when the corpus callosum bridges across the two hemispheres (approximately Bregma +1.10 mm).

#### Stereology of NeuN-stained sections

Unbiased stereological estimates of volume and neuronal number were obtained using StereoInvestigator software (Microbrightfield, Willston, VT). All stereological measurements were performed with the observer being blinded to the experimental grouping. The Cavalieri method was used to obtain unbiased estimates of striatal and M1 cortical reference volumes [45]. ROIs were defined by x1.6 magnification lens through reference to neuroanatomical landmarks. For both the striatum and M1 cortex, equally spaced sections (50 μm thickness each, typically 4-6 sections with a 450 μm gap) were analyzed (see Figure S1 for boundaries drawn on histological sections).

As defined by Sadikot and Sasseville [46], for striatal measurements, sections were sampled anteriorly from the first appearance of the genu of the corpus callosum (Bregma = +1.1 mm) to the first evidence of a hippocampal formation posteriorly (Bregma = -0.94 mm). The dorsal and lateral boundaries consisted of the corpus callosum with the medial boundary being the lateral ventricles/external capsule. For sections rostral to where the dorsal 3^rd^ ventricle has joined the lateral ventricles, ventral boundaries become lateral ventricles/globus pallidus. The striatal volume was sampled on 4-5 sections for both WT and R6/1. 

M1 cortex was measured anteriorly from +1.1mm Bregma to posteriorly -0.94 mm Bregma, from layers II to VI, as defined in the stereotaxic atlas [47]. The absence of cortical layer IV (indicative of the S1 cortex) defined the lateral boundaries of M1, whereas medial boundaries consisted of the most dorsal part of the corpus callosum. M1 was sampled on 4-5 sections for both WT and R6/1.

To obtain unbiased estimates of neuronal numbers, the optical fractionator was employed as a stereological probe (coefficient of error <0.1). Section thickness and neuronal counts were performed under oil immersion with the x100 objective (Zeiss) with a numerical aperture of 1.4. A sampling grid was applied appropriate to the structure measured (cortex = 200 μm x 200 μm, striatum = 400 μm x 400 μm) with a counting frame of 65 μm x 35 μm with a mean thickness of 18 μm. Guard zones of 0.5 μm were applied at the top and the bottom of each frame with a mean dissector height of 17 μm. 

#### Quantitative analysis of S830

Evaluation of mHTT immunoreactivity in different brain regions was performed using an intensity-based measurement of S830 staining. Non-overlapping images (using fixed exposure and light intensities at x40) were obtained from 3 consecutive sections expressing the striatum or hippocampus, and 6 sections for the cortex. In total, 30 striatal (10 per section), 60 cortical (10 per section) and 36 hippocampal subregion (12 per section) images were taken. All images were captured in RGB using a live video camera (JVC, 3CCD, KY-F55B), mounted onto a Zeiss Axioplan microscope.

Staining intensity was quantified using threshold-based analysis software (Image Pro Plus, Media Cybernetics, IL, USA) assessing optical density of the immunoreactive product. Threshold levels were chosen based on the minimum level of transmitted light needed to detect the immunoreactive product on a scale of 0 (100% transmitted light) and 255 (0% transmitted light) for each pixel. Two levels were taken to measure dense, nuclear mHTT inclusions (90), and total aggregated mHTT staining (nuclear and extra-nuclear, 130); mean percentage immunoreactivity area per field of view (FOV) was recorded.

### Statistical analyses

Data was graphed using Prism Ver.5.0b (GraphPad Software, California, USA). Statistical analyses were calculated using SPSS Statistics Ver.20 (IBM, Portsmouth, UK). All data were screened for statistical outliers using Grubbs’ Test (GraphPad Software, California, USA). Grubbs’ test calculates a z ratio for each value within a given dataset (determined through subtracting each value from the group mean, and dividing it by the standard deviation). If the z ratio for any given value is greater than those proposed by Grubbs for that population size, it is considered an outlier. If this was the case, the value was excluded from analysis. Due to either animal loss (total of 2 cases, female R6/1 only), or occasional missing data samples and removal of statistical outliers (total absent data samples for male WT = 15, male R6/1 = 8, female WT = 8, female R6/1 = 20), the number of animals varied for each test at different time points. A table summarizing the number of subjects included for each measure and the reason for exclusion can be found in Table S1. 

The acquired datasets can be separated into those that were collected longitudinally, those acquired at a single time point, correlational analyses, as well as Tensor Based Morphometry statistics. 

#### Longitudinal datasets

These datasets refer to tests where data was collected at more than one time point and include: body weight assessment, body temperature assessment, locomotor activity, grip strength measurements, behaviors in a swimming T-maze and measurements of volumetry and T2 relaxivity through MRI. It was not possible to compute a repeated measures ANOVA due to missing values at different measurement times. To probe the influence and interaction of genotype and gender across time, a three-way ANOVA was applied (Genotype, Gender and Age, as between-subject factors). To determine whether mice exhibit cued fear conditioning extinction (i.e. a form of re-learning that the conditioned stimulus is no longer aversive with repeated exposure), a repeated measures ANOVA was used (repeated Tone (conditioned stimulus) exposure as within-subject factor, Genotype and Gender as between-subject factors). Post-hoc tests with a Bonferroni Correction for multiple comparisons were applied where appropriate. All main effects from ANOVAs can be found in Table S2.

#### Datasets taken at a single time-point

These data refer to tests that were only conducted once during the study and include: rotarod, odor discrimination, social interaction, fear conditioning, and histological measures taken through stereology. To probe the influence of genotype and gender on these measures, a two-way ANOVA was applied (Genotype and Gender as between-subject factors). Post-hoc tests with a Bonferroni Correction for multiple comparisons were applied where appropriate. All main effects from ANOVAs can be found in Table S2.

For the histological quantification of mHTT, there was an absence of immunoreactivity in the WT mice. This marker, within this group, was therefore not included in the analysis. To compare levels of mHTT across various brain regions, and probe the influence of gender on this measure a two-way ANOVA was applied (Region and Gender as between-subject factors). A post-hoc test was conducted for multiple comparisons, with a Bonferroni Correction, comparing male and female R6/1 mice at each brain region.

#### Correlative analyses

The Pearson Correlation Coefficient was used for all correlative analyses. To account for multiple correlations of the same data, the significance level was corrected using a Bonferroni Correction by dividing the standard p value (0.05) by the number of comparisons made, resulting in an appropriately adjusted p value for each dataset.

#### Tensor Based Morphometry Statstics

As described in [39], a non-parametric two-tailed *t*-statistic, assuming unequal variance between groups, is computed at each voxel (approximately 42,000) in the brain; permutation-testing is used to assess significance. The effective number of permutations at each voxel is increased by pooling null-distribution statistics from other voxels to allow for accurate multiple comparisons correction using the False Discovery Rate [48]. The minimum number of required permutations is approximately (number-of-voxels / FDR-significance-level) = 42,000 / 0.05 = 840,000.

## Results

### Physiological measures

Abnormal weight gain is a common feature of mouse models of HD. Both male (Figure 1B) and female (Figure 1C) R6/1 mice had lower body weights at later ages compared to WTs [F(Genotype X Age)_4,195_ =6.173, p<0.001]. Although there was no interaction between gender and genotype [F(Genotype X Gender)_1,195_=0.845, ns] weight loss reached statistical significance for male mice at 19 weeks only (p=0.025, multiple comparison with Bonferroni Correction). Overall, differences in body temperature were detectable between WT and R6/1 mice (data not shown; [F(Genotype)_1,157_=5.768, p=0.018]), but there was no interaction with gender [F(Genotype X Gender)_1,157_=0.18, ns] or age [F(Genotype X Age)_3,157_=0.293, ns]. Moreover, upon post-hoc testing, when corrected for multiple comparisons, there were no genotype-dependent differences detected at any given time point.

### R6/1 exhibit age-related motor and cognitive deficits

The presence of motor abnormalities is an important feature of HD mouse models, here we tested WT and R6/1 mice at three tasks designed to probe motor ability: rotarod, locomotor activity and grip strength. 

#### Motor-related-tasks

**Figure 2:**
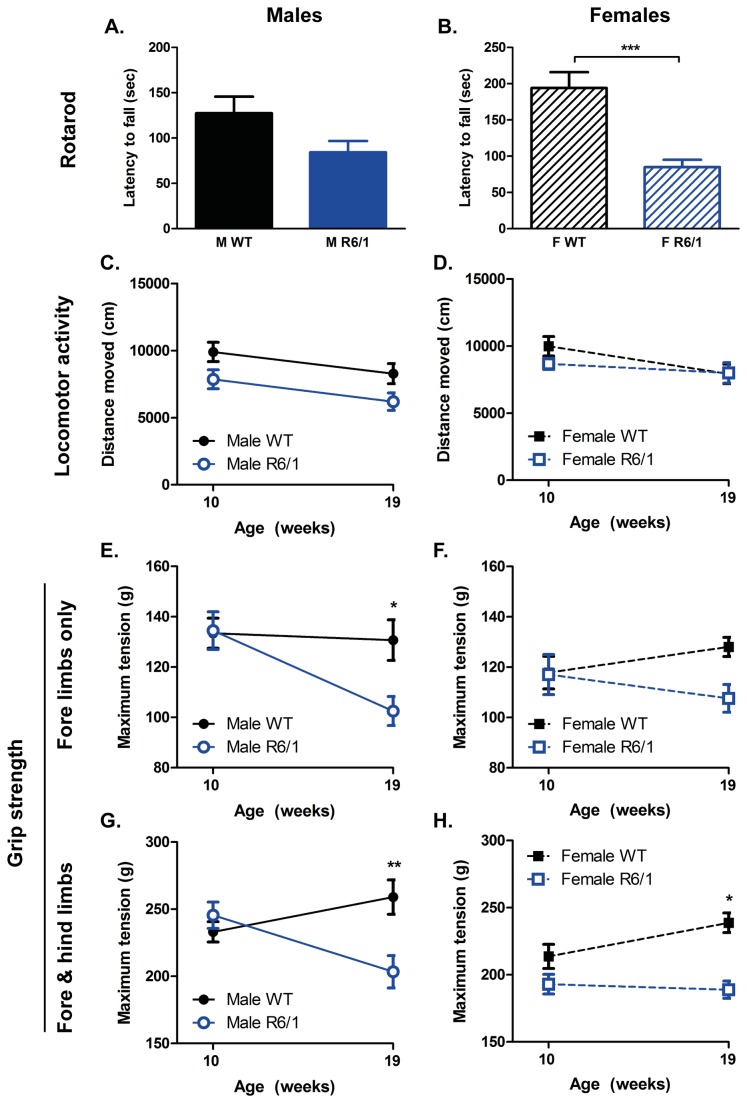
Performance at motor-related tasks. Both male (A) and female (B) R6/1 mice exhibited lower latency to fall from an accelerating rotarod at 11 weeks of age, reaching statistical significance in the females only. Conversely there was no difference in locomotor activity in an open field between WT and R6/1 mice at either 10 or 19 weeks of age (C & D). Forelimb grip strength (E & F) declined with age in R6/1 mice, as did both fore- and hind limb strength when taken together (G & H). Rotarod analyzed using two-way ANOVA (Genotype X Gender); locomotor activity and grip strength analyzed using three-way ANOVA (Genotype X Gender X Age). Rotarod: WT male n=11, R6/1 male n=9, WT female n=10, R6/1 female n=10; Locomotor activity: WT male n=11, R6/1 male n=9, WT female n=10, R6/1 female n=8-9; Grip strength: WT male n=11, R6/1 male n=9, WT female n=10, R6/1 female n=8-10. All data presented as means ± SEM; *p<0.05, **p<0.01, ***p<0.001.

Rotarod was tested at 11 weeks of age only. There was an overall difference between the two genotypes [F(Genotype)_1,39_=20.794, p<0.001], but no interaction with gender [F(Genotype X Gender)_1,39_ =3.918, ns]. Despite having a lower performance compared to WTs at this task, male R6/1s did not reach statistical significance (Figure 2A), whereas, female R6/1s were significantly impaired versus WTs (p<0.001, Figure 2B). 

Exploratory activity in the open field was assessed during the dark cycle, at both 10 and 19 weeks of age. Overall, WT and R6/1 mice behaved differently during this task [F(Genotype)_1,76_=7.296, p=0.009], but there was no interaction with either gender [F(Genotype X Gender)_1,76_=2.115, ns] or age [F(Genotype X Age)_1,76_=0.443, ns]. After correcting for multiple comparisons, exploratory activity exhibited by either male (Figure 2C) or female (Figure 2D) R6/1 mice was not different to WT controls at either age studied. 

Grip strength capacity (forelimbs; simultaneous fore- and hind limbs) was assessed at both 10 and 19 weeks of age. Forelimb grip strength capacity of R6/1 mice changed over time [F(Genotype X Age)_1,77_=6.712, p=0.012], but this was not influenced by gender [F(Genotype X Gender)_1,77_=0.1, ns]. Maximum tension exhibited by both male (Figure 2E) and female R6/1s (Figure 2F) dropped with age, with only males being statistically different at 19 weeks (p=0.015). The grip strength of the fore- and hind limbs, when taken together, followed a similar pattern to that of the forelimbs only, as there was an age-related change in performance between WTs and R6/1s [F(Genotype X Age)_1,77_=13.107, p=0.001], and, again, gender did not exert a significant influence [F(Genotype X Gender)_1,77_=1.033, ns]. Maximum tension of the fore and hind limbs of the R6/1s dropped to below that of WTs at 19 weeks of age for both males (p=0.003, Figure 2G) and females (p=0.016, Figure 2H).

#### Non-motor-related tasks

Clinical signs of HD are not restricted to motor abnormalities and these features should also be recapitulated in mouse models for greater face validity. To probe such non-motor behaviors, mice were exposed to learning and memory paradigms (swimming T-maze and fear conditioning), as well as measures of olfactory function (odor discrimination) and recognition of social novelty (social interaction).

**Figure 3:**
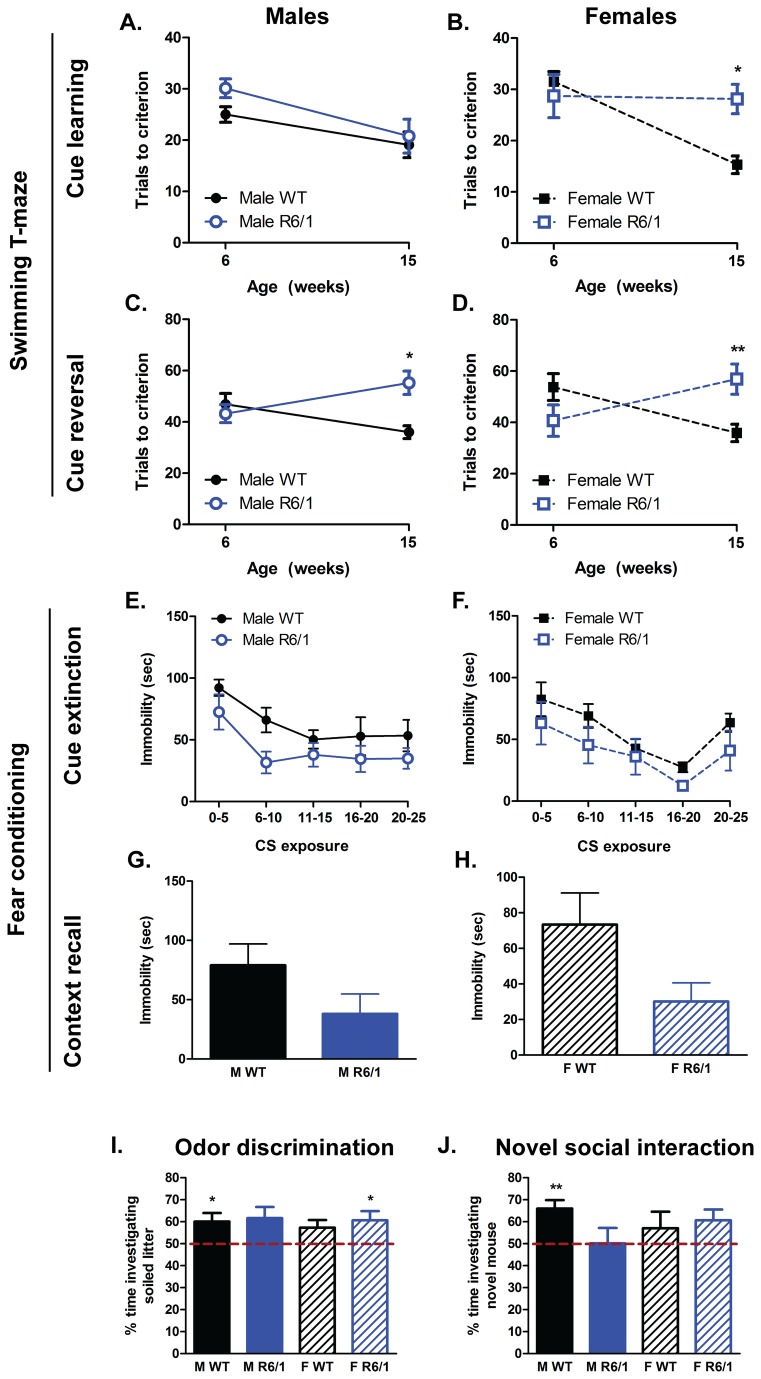
Performance at non-motor-related tasks. Male R6/1 mice did not exhibit any impairment in cue learning in the swimming T-maze (A), whereas female R6/1 mice were deficient at 15 weeks of age (B). Both male (C) and female (D) R6/1s developed an age related deficit in cue reversal learning in a swimming T-maze. There was no obvious difference in cued extinction behavior between WTs and R6/1s (E & F), or a difference in contextual recall (G & H). At the odor discrimination task (I) and novel social interaction test (J) there was no difference between the four groups. Swimming T-maze analyzed using three-way ANOVA (Age X Genotype X Gender); fear conditioning cue extinction analyzed using a repeated measures ANOVA (Genotype X Gender X Tone); fear conditioning context recall, odor discrimination and novel social interaction analyzed using a two-way ANOVA (Genotype X Gender). Swimming T-maze: WT male n=10-11, R6/1 male n=8-9, WT female n=10, R6/1 female n=9-10; Fear conditioning: WT male n=11, R6/1 male n=9, WT female n=7-8, R6/1 female n=7-8; Odor discrimination: WT male n=11, R6/1 male n=8, WT female n=9, R6/1 female n=8; Novel social interaction: WT male n=10, R6/1 male n=9, WT female n=10, R6/1 female n=10. All data presented as means ± SEM; *p<0.05, **p<0.01.

 The swimming T-maze was used for the assessment of cued learning and its subsequent reversal at both 6 and 15 weeks of age. Overall, the ability to learn a visual cue signifying an escape route (termed “cue learning”) was different between WT and R6/1 mice [F(Genotype)_1,77_ =5.066, p=0.028]. Despite there not being a significant interaction with either gender [F(Genotype X Gender)_1,77_=0.185, ns] or age [F(Genotype X Age)_1,77_=2.663, ns], male R6/1s were not significantly different to WT controls at either age (Figure 3A), but female R6/1 mice took longer to learn the rule compared to female WTs at 15 weeks only (p=0.01, Figure 3B). Reversing the previously learned cue-based rule, termed “cue reversal”, is a more cognitively demanding task and this was reflected by the generally longer trials required to reach criterion (on average 46.01% longer compared to the more simple cue learning). There was an age-related difference in cue reversal learning between WT and R6/1 mice [F(Genotype X Age)_1,75_=19.17, p<0.001]. Similar to the cue learning phase of this task, gender did not influence behavior at cue reversal [F(Genotype X Gender)_1,75_=0.348, ns]. Post-hoc analyses revealed that at 6 weeks of age R6/1 mice had a similar learning capacity to WTs, but by 15 weeks both male (p=0.018, Figure 3C) and female R6/1s (p=0.006, Figure 3D) took longer to learn this rule compared to WT controls. 

 Fear conditioning relies upon the formation of a conditioned fear response to an auditory tone and salient context, and provides a measure of memory retention and extinction. Performance at this task was investigated at 12 weeks of age in WTs and R6/1s. On the training day, immediately following the conditioning foot-shock exposure, there was no difference in immobility (fear expression) between the WT and R6/1 mice (data not shown; [F(Genotype)_1,35_=0.193, ns]) and there was no influence of gender [F(Genotype X Gender)_1,35_=0.436, ns], indicating a similar response to the conditioning protocol, and, hence, memory acquisition. On the second day, all animals were subjected to repeated exposure of the conditioning tone without foot-shock administration to probe memory performance. Initially mice expressed a robust fear response, and, with repeated exposure to the tone, mice exhibited less immobility ([F(Tone)_4,27_=6.879, p=0.001]; Figure 3E&F), indicative of cue extinction learning. There was no interaction between Genotype and the response to the repeated Tone [F(Genotype X Tone)_1,27_=1.116, ns] suggesting a similar pattern of extinction learning between WTs and R6/1s. However, overall, fear response across all these tones was significantly lower in R6/1s compared to WTs [F(Genotype)_1,30_=6.824, p=0.014], indicating some abnormality in R6/1 performance at this task, but there was no influence of gender on this behavior [F(Genotype X Gender)_1,30_=0.042, ns]. On the third day of the task, contextual recall was tested over 5 min. Overall R6/1 mice expressed less fear response compared to WTs ([F(Genotype)_1,34_=6.006, p=0.02]; Figure 3G&H), but this was not influenced by gender [F(Genotype X Gender)_1,34_=0.004, ns]. These results imply an impaired memory performance in the R6/1 mice at 12 weeks, but the exact nature of the deficit remains unclear. 

Olfaction was tested in the mice at 10 weeks of age using the odor discrimination task (Figure 3I). There was no difference in WT and R6/1 at the performance of this task [F(Genotype)_1,35_=0.349, ns] and no effect of gender [F(Gender)_1,35_ =0.204, ns]. Following the odor discrimination task, mice were tested at their social interaction and ability to determine novelty at 10 weeks. On the first part of the task, time spent investigating an unfamiliar mouse was similar between WTs and R6/1s [F(Genotype)_1,39_=0.936, ns], which would suggest unaffected social behaviors in the R6/1s. At the second part of the task (Figure 3J), (exposure to the “familiar” mouse from the first trial along with a “novel mouse”), there was no difference in preference for novelty between WT and R6/1 [F(Genotype)_1,38_=0.253, ns], and no influence of gender [F(Gender)_1,38_=0.704, ns].

**Figure 4:**
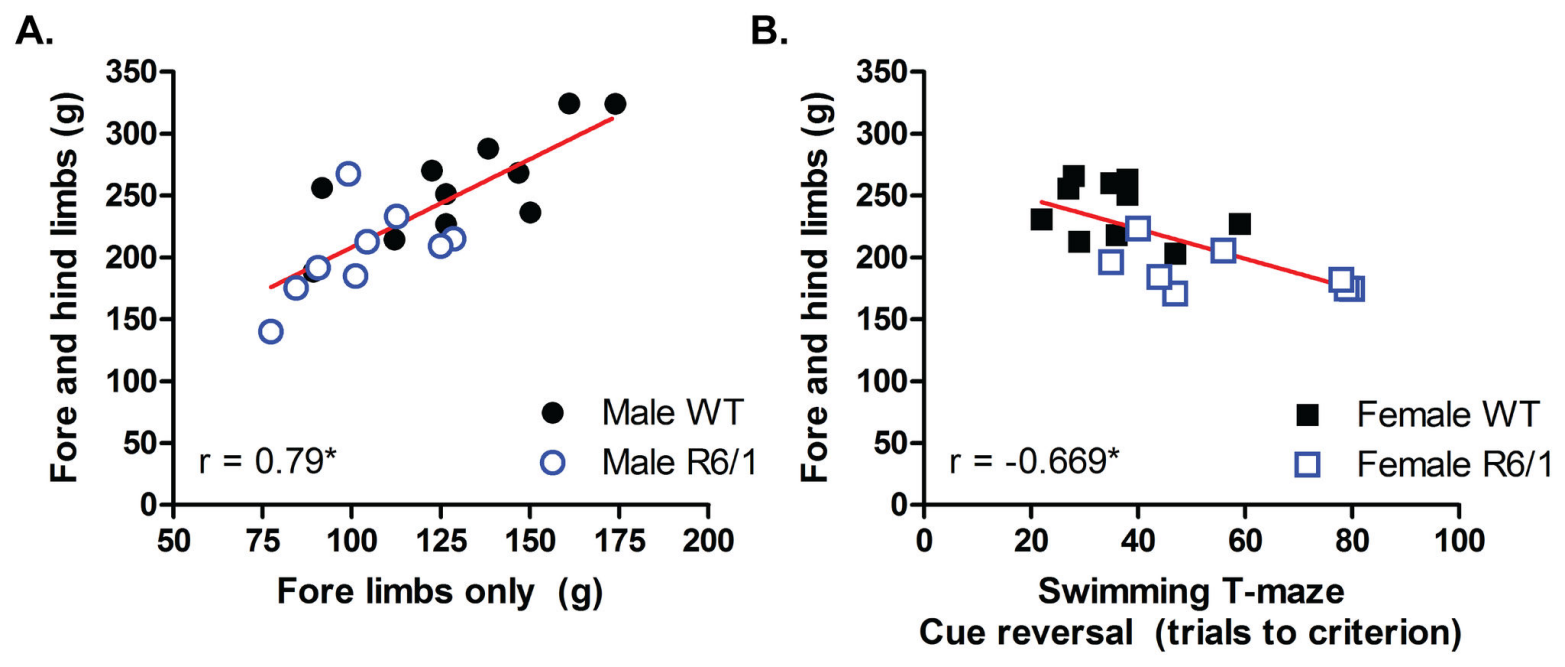
Concomitant deficits in behavioral tasks result in a robust correlation of those two measures. (A) At 18 weeks of age R6/1 mice have both lower forelimb, and fore- and hind limb grip strength capacity to a similar degree, separating the data from those of WT resulting in a significant, positive correlation (r=0.79, p<0.005). (B) Between 15-19 weeks of age R6/1 mice have lower fore- and hind limb grip strength, as well as impaired cue reversal learning in a swimming T-maze to a similar degree, forming a strong overall negative correlation (r=-0.669, p<0.005).

To determine interactions and associations of performance at these behavioral tasks (both motor- and non-motor-related), a correlative analysis was performed. These analyses were separated by age tested; behaviors measured between 6-12 weeks (Table S3) and behaviors measured between 15-19 weeks (Table S4). There were no significant correlations between the performance at the various behavioral tasks recorded between 6 and 12 weeks of age. However, at 15 to 19 weeks, a higher grip strength in the forelimbs only was associated with higher grip strength in both the fore- and hind limbs for the males (r=0.79, p<0.005). A longer time to learn the cue reversal task in the swimming T-maze was only associated with lower fore- and hind limb grip strength in females (r=-0.669, p<0.005). These effects were only evident, however, when considering both WT and R6/1 mice together, and, therefore likely reflect the concomitant development of impairments in two measures in R6/1 mice; as performance in two measures declines over time, a strong correlation is formed (Figure 4A&B). 

### R6/1 exhibit progressive brain atrophy

**Figure 5:**
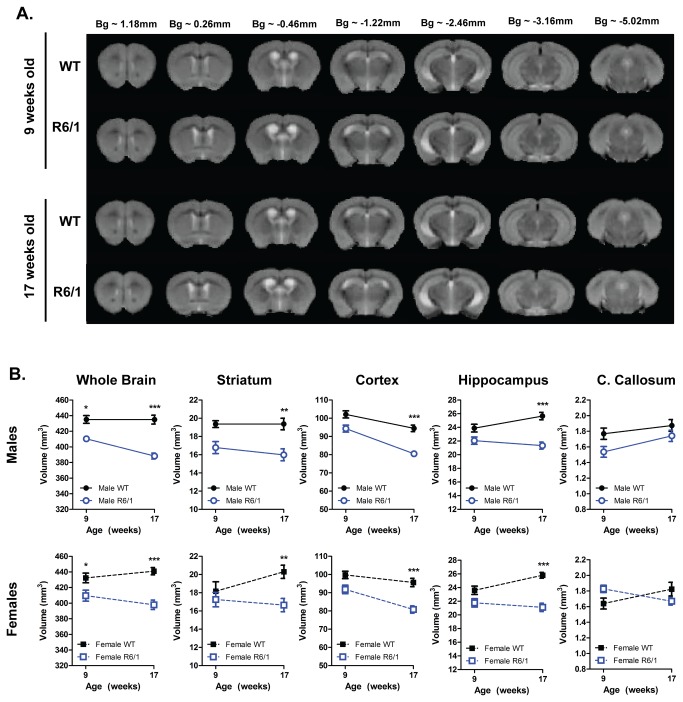
Longitudinal *in vivo* MRI. (A) Summed T2-weighted structural group-images from WT and R6/1 mice at 9 and 17 weeks of age. (B) R6/1 mice progressive lost regional brain volumes compared to WT controls, with the exception of the corpus callosum (C. Callosum). Analyzed using a three-way ANOVA (Genotype X Gender X Age). Striatum, cortex, hippocampus, corpus callosum and whole brain: WT male n=11, R6/1 male n=9, WT female n=10, R6/1 female n=9-10. All data presented as means ± SEM; *p<0.05, **p<0.01, ***p<0.001.

Alongside the detailed analysis of behavioral dysfunction in R6/1 mice, longitudinal *in vivo* MRI was conducted at 9 and 17 weeks of age to investigate changes in neuroanatomy (Figure 5A). The striatum, hippocampus, cortex, corpus callosum and whole brains were manually delineated and regional volumetry and T2 relaxivity were measured in the same ROIs. 

Longitudinal MR volumetry provides a measure of volume change in the R6/1 mouse brain (Figure 5B). The whole brain volume of R6/1 mice was different to WTs over time [F(Genotype X Age)_1,78_=7.501, p=0.008]. In fact, R6/1s were already 94.5% that of the WTs at 9 weeks of age, reaching statistical significance for both the males (p=0.023) and females (p=0.044), although there was no overall interaction between genotype and gender [F(Genotype X Gender)_1,78_=0.138, ns]. This difference was amplified at 17 weeks to 89.79%. Overall striatal [F(Genotype)_1,78_=26.481, p<0.001], cortical [F(Genotype)_1,78_=61.667, p<0.001] and hippocampal volumes [F(Genotype)_1,78_=64.687, p<0.001] were different between WT and R6/1 mice, but this was not influenced by gender (striatum [F(Genotype X Gender)_1,78_=0.464, ns]; cortex [F(Genotype X Gender)_1,78_=0.033, ns]; hippocampus [F(Genotype X Gender)_1,78_=0.056, ns]). There was a significant interaction between genotype and age in the cortex [F(Genotype X Age)_1,78_=5.224, p=0.025] and hippocampus [F(Genotype X Age)_1,78_=11.447, p=0.001], but, interestingly, this interaction was not evident for the striatum [F(Genotype X Age)_1,78_=3.019, ns]. A Bonferroni-corrected multiple comparison analysis revealed no differences in these regional volumes at 9 weeks of age, but by 17 weeks of age all three regions were significantly smaller in the R6/1s: the R6/1 striatum was 82.36% that of WT (males p=0.008, females p=0.004); the R6/1 cortex was 84.91% that of WT (males p<0.001, females p<0.001); the R6/1 hippocampus was 82.49% that of WT (males p<0.001, females p<0.001). There was no detectable difference in corpus callosum volume at either 9 or 17 weeks of age. Whole brain volume was significantly correlated with cortical (r=0.708, p<0.0011) and hippocampal (r=0.693, p<0.0011) volumes in female R6/1s (Table S5), suggesting a relationship between changes in whole brain and sub-regional volumes.

 As well as measures of brain volumes, T2 relaxivity measures (reflective of tissue composition) were taken in the striatum, cortex, hippocampus, corpus callosum and muscle tissue. There were few differences in regional T2 values between WT and R6/1 mice (data not shown). Overall, T2 relaxation times were different between WTs and R6/1s in the striatum [F(Genotype)_1,77_=13.294, p=0.001] and corpus callosum [F(Genotype)_1,76_=4.403, p=0.04] only, there were no interactions between genotype with age or gender in any region studied. A post-hoc analysis revealed only a shortening of T2 values between male WT and R6/1 mice at 9 weeks in the striatum (p=0.002), cortex (p=0.009) and hippocampus (p=0.039). There were no significant effects in the corpus callosum or muscle tissue. 

 Compared to the volumetric data, T2 values across the various brain regions studied were highly correlated with each other for both WTs and R6/1s, irrespective of gender (Table S5). This, however, was not the case for the corpus callosum or muscle tissue. These data indicate that changes in T2 values are highly consistent and ubiquitous across grey matter irrespective of mouse genotype. There were no significant correlations between measures of volumetry and T2 relaxivity with the exception of a smaller whole brain volume being associated with higher T2 relaxation times in the male R6/1s only (r=-0.756, p<0.0011).

 The neuroanatomical basis of behavioral impairments is reflected in associations between these two outcome measures, thus a correlative approach to probe associations between brain abnormalities detected through MRI and age-matched functional impairments in behavioral tasks was taken between early (between 6 and12 weeks) and later measures (15 and 19 weeks) (Table S6). Only two significant associations were detected after corrections for multiple comparisons, both in early measures of behavior and MRI (6 to 12 weeks). When considering both WTs and R6/1s together, longer hippocampal T2 relaxation was associated with hyperactivity in an open field (r=0.702, p<0.001), evident in the males only. Moreover, longer hippocampal T2 was also associated with a lower immobility (memory recall) in the cued fear conditioning task (r=-0.761, p<0.001), but in the females only.

### TBM analysis revealed sub-region specific and gender-dependant differences in the R6/1 brain atrophy

**Figure 6:**
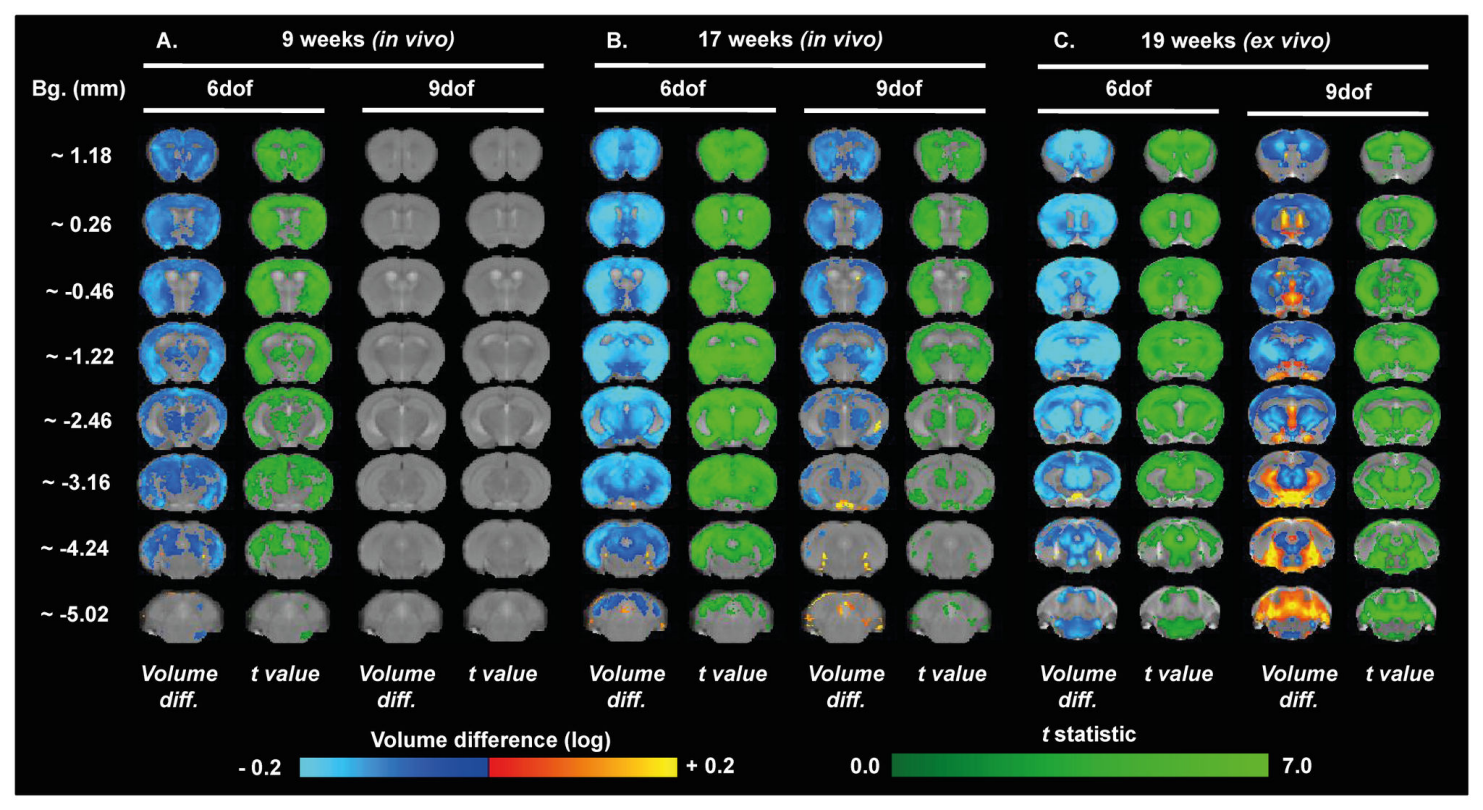
Brain volume changes in male WT and R6/1 assessed through TBM. Maps of local volumetric changes in male R6/1 mice compared to male WT 9 weeks *in* *vivo* (A), 17 weeks *in* *vivo* (B), and at 19 weeks *ex* *vivo* (C). Images presented as either both global volume change (6 dof), or region-specific changes corrected for global change (9 dof). Color scales are for volume difference (Volume diff., blue-to-yellow), and the raw *t* statistical value at each voxel (dark-to-light green). Only volume changes and effects-sizes which survive multiple comparisons corrections across all voxels in the brain (False Discovery Rate with q<0.05) are shown.

**Figure 7:**
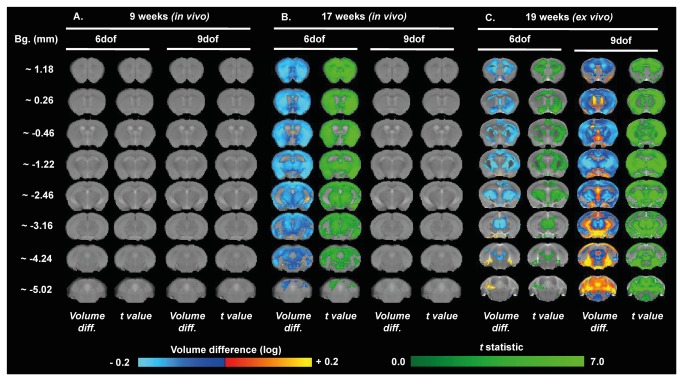
Brain volume changes in female WT and R6/1 assessed through TBM. Maps of local volumetric changes in female R6/1 mice compared to female WT 9 weeks *in* *vivo* (A), 17 weeks *in* *vivo* (B), and at 19 weeks *ex* *vivo* (C). Images presented as either both global volume change (6 dof), or region-specific changes corrected for global change (9 dof). Color scales are for volume difference (Volume diff., blue-to-yellow), and the raw *t* statistical value at each voxel (dark-to-light green). Only volume changes and effects-sizes which survive multiple comparisons corrections across all voxels in the brain (False Discovery Rate with q<0.05) are shown.

Although informative, manually segmented ROI analysis has some limitations, such as a lack of sub-regional specificity and the selective choice of regions to be studied. Tensor Based Morphometry (TBM), an automated image analysis, allows for the unbiased detection of changes in individual voxels across the whole brain irrespective of their ROI assignment. TBM was used to compare WT and R6/1 brains at three time points scanned, 9 weeks and 17 weeks *in vivo*, and 19 weeks *ex vivo* for males (Figure 6) and females (Figure 7). Although there was evidence of global brain volume shrinkage (6 dof) in male R6/1 mice at 9 weeks (Figure 6A), but not female (Figure 7A), there was no specific regional differences when corrected for whole brain volume change for either gender (9 dof). By 17 weeks there was a substantial shrinkage of both whole brain volume (Figure 6B), as well as sub-regions when corrected for global volume change in male mice only. Specifically, these analyses revealed subtle expansion of posterior ventricular spaces and, interestingly, in R6/1s, a spared hippocampal volume. Similar to that detected through the manually segmented analysis, the TBM analysis also indicates that the male R6/1 cortex suffered atrophy, but this advanced technique allows for a more precise description of this change, as it is apparent that TBM has revealed a global cortical atrophy, but with a relative sparing of the cingulate cortex (at Bregma ~0.26-0.46 mm). These TBM analyses provide more precise evidence of sub-regional changes that occur within large brain regions, such as the cortex. 

Female R6/1s had robust global volume differences (6 dof), but no sub-regional changes (9 dof) were evident even at 17 weeks (Figure 7B). These effects are similar, but more robust and better defined, on the analysis of the *ex vivo* images taken at 19 weeks, where differences between male (Figure 6C) and female mice (Figure 7C) were less evident. Interestingly, for both genders, the greatest magnitude of shrinkage was evident in the posterior striatum at Bregma ~ -1.22 mm, and substantial cortical atrophy was evident, specifically in the retrosplenial areas, visible at Bregma ~ -2.46 mm. 

**Figure 8:**
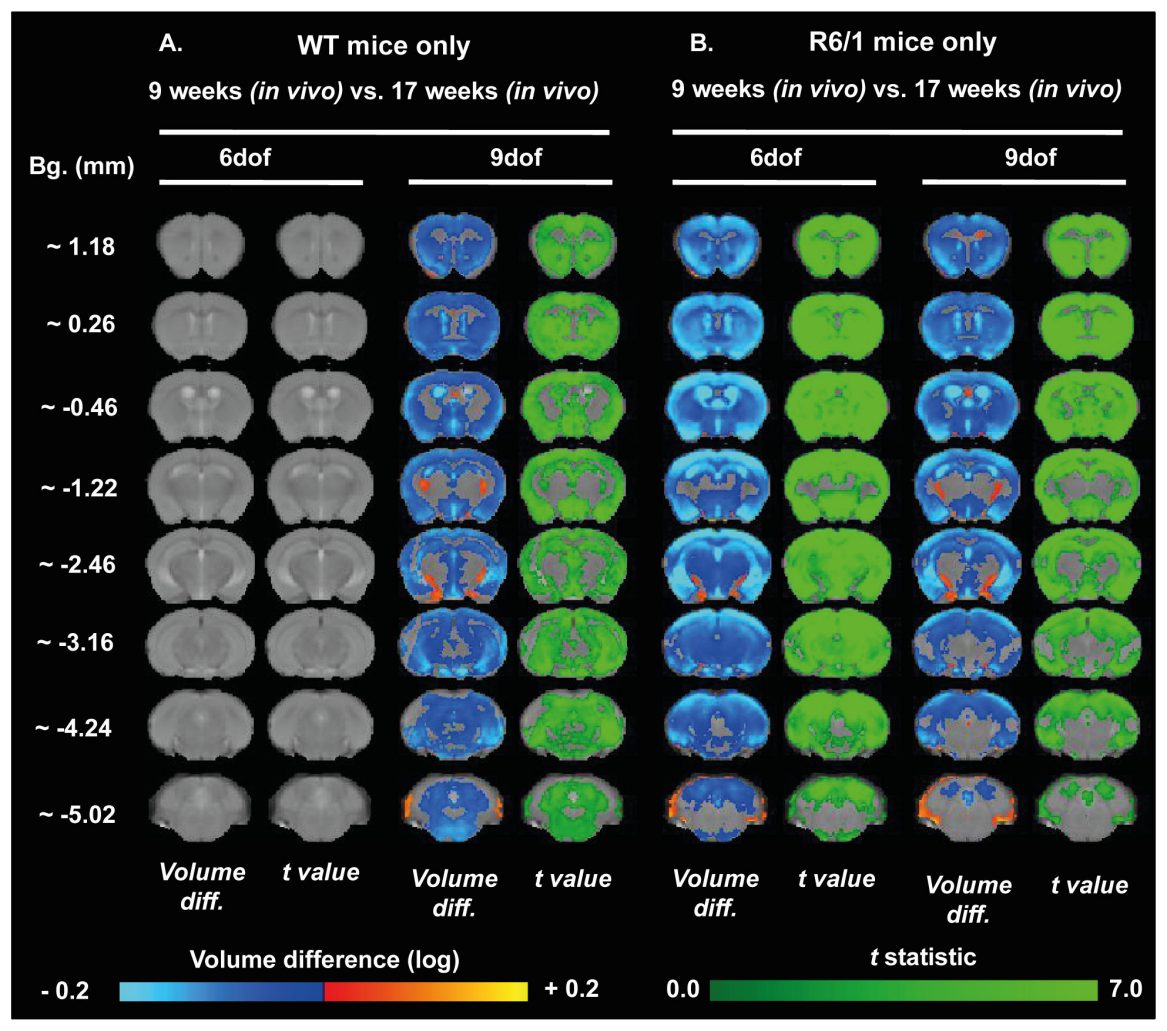
Age-related evolution of brain atrophy in male mice measured through TBM. Maps of local volumetric change in both male WT (A) and male R6/1 mice (B) between the two time points (9 and 17 weeks of age). Images presented as either global volume change (6 dof) or regional-specific changes corrected for global change (9 dof). Color scales are for volume difference (Volume diff., blue-to-yellow) and the raw *t* statistical value at each voxel (dark-to-light green). Only volume changes and effects-sizes which survive multiple comparisons corrections across all voxels in the brain (False Discovery Rate with q<0.05) are shown.

**Figure 9:**
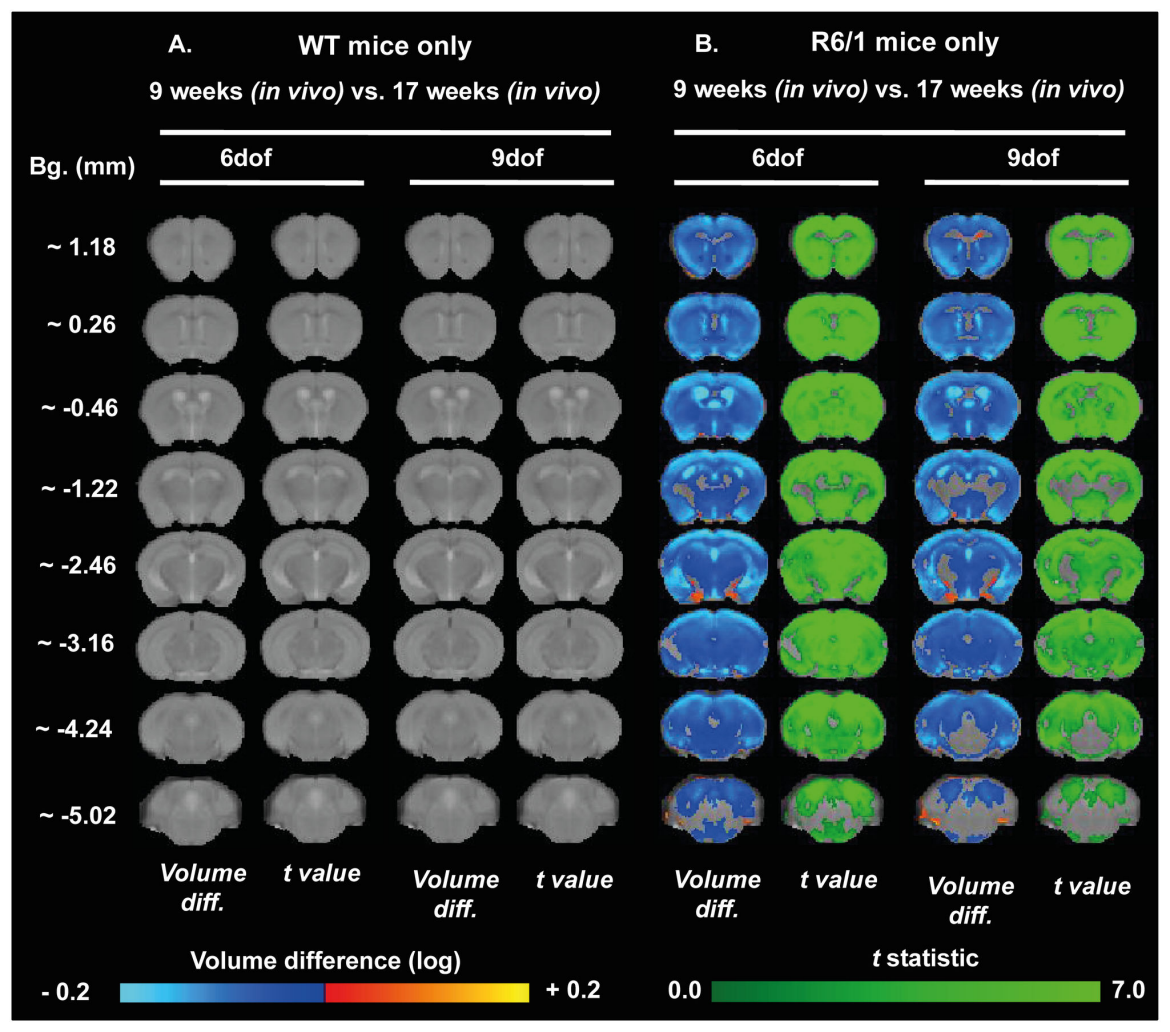
Age-related evolution of brain atrophy in female mice measured through TBM. Maps of local volumetric change in both female WT (A) and female R6/1 mice (B) between the two time points (9 and 17 weeks of age). Images presented as either global volume change (6 dof) or regional-specific changes corrected for global change (9 dof). Color scales are for volume difference (Volume diff., blue-to-yellow) and the raw *t* statistical value at each voxel (dark-to-light green). Only volume changes and effects-sizes which survive multiple comparisons corrections across all voxels in the brain (False Discovery Rate with q<0.05) are shown.

In order to determine specific age-related alterations within each separate genotype, a longitudinal analysis was conducted to compare these across time for both male (Figure 8) and female (Figure 9) mice. Unexpectedly, male WT mice had no global brain volume changes over time, but did show some evidence of specific sub-regional brain changes, principally a shrinkage of the anterior striatum and a ubiquitous cortical atrophy, whist posterior ventricular spaces were enlarged, (Figure 8A), whereas there were no age-related differences in female WT mice (Figure 9A). Both male (Figure 8B) and female (Figure 9B) R6/1 mice exhibited global brain volume change and specific regional changes when corrected for whole volume change. Indeed, both male and female R6/1 mice exhibited a ubiquitous shrinkage of the striatum over time, whereas, within the cortex, the somatosensory cortices were dramatically atrophied at Bregma ~1.22 mm. Interestingly, for both WT and R6/1 mice, the corpus callosum remained relatively unchanged with age, consistent with manually segmented ROIs. 

In summary, both methods of measuring volume change from MRI (i.e. manually segmented volumetry and automated TBM) detected robust shrinkage of grey matter tissue, and a relative sparing of white matter tissue, in R6/1 mice with age. Nevertheless, TBM allowed for a more precise description of the anatomical loci of these changes.

### R6/1 mice express HD-like histopathological features, including neuronal loss

**Figure 10:**
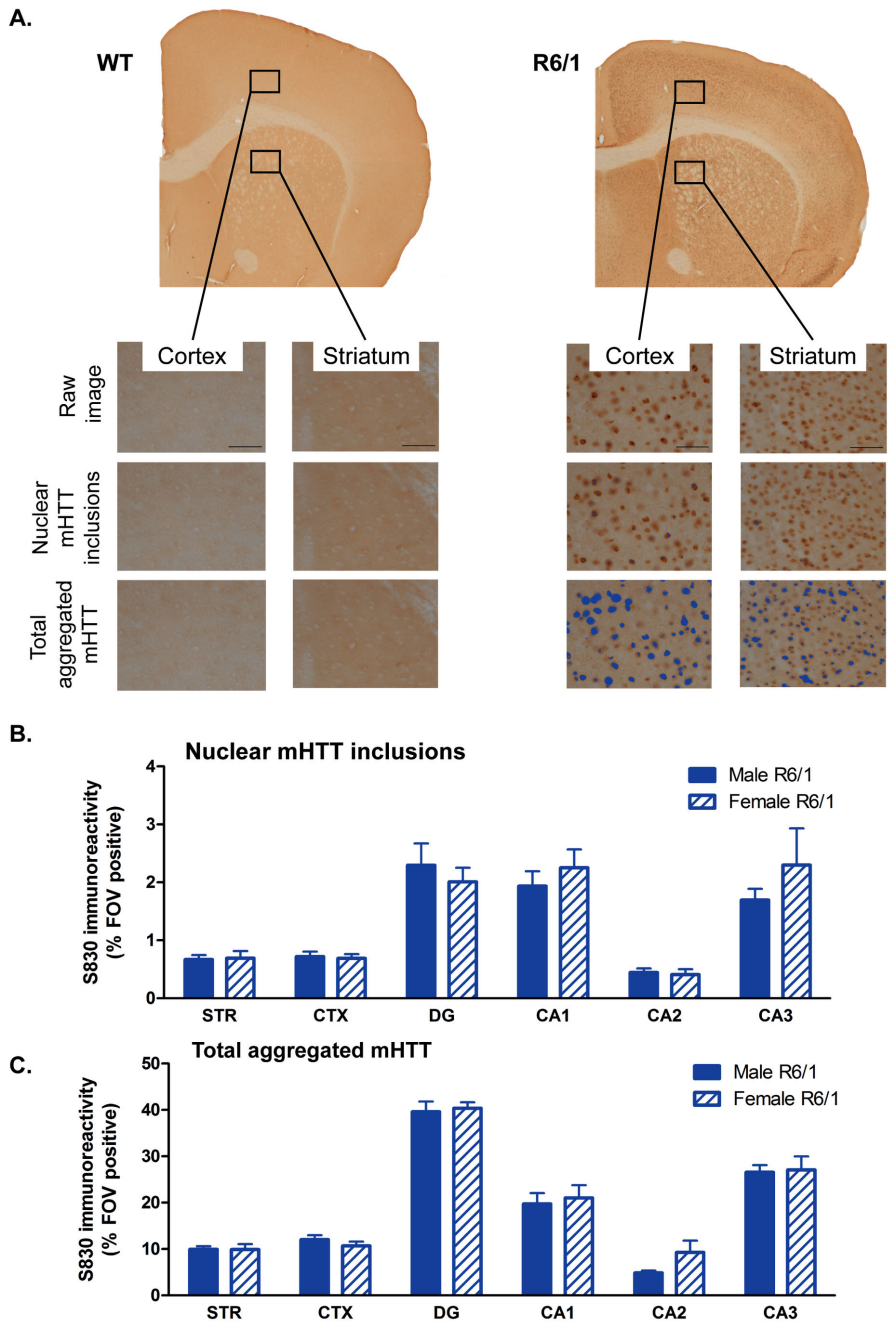
mHTT accumulation in R6/1 mice. (A) Representative coronal sections from 19 weeks’ old WT and R6/1 mouse brains stained with the S830 antibody for the detection of mHTT. Levels were quantified within seven brain regions using an intensity threshold-based image analysis tool optimized for the detection of nuclear inclusions, or total levels of aggregated mHTT (neuropil aggregates and diffuse nuclear accumulation, highlighted in blue); scale bar 50μm. Field of views (FOVs) were sampled in the cortex, striatum and hippocampal subfields. Percentage of FOVs positive for S830 stain of nuclear inclusions (B), and percentage of FOV positive for total aggregated mHTT (C), there were no significant differences between male and female R6/2 for either assessments. Regional differences in mHTT reflect cellular density. STR = striatum, CTX = cortex, DG = dentate gyrus, CA1 = hippocampal CA1 subfield, CA2 = hippocampal CA2 subfield, CA3 = hippocampal CA3 subfield. Analyzed using a two-way ANOVA (Gender X Region). All brain regions investigated: R6/1 male n=8-9, R6/1 female n=7-9. Data presented as means ± SEM.

In order to determine the molecular and neurological basis for the behavioral dysfunction and brain atrophy observed through MRI, a histopathological investigation was conducted. The deposition of mHTT is a neuropathological hallmark of HD. We used the S830 antibody to independently visualize nuclear mHTT inclusions, as well as other forms of aggregated mHTT in both the nucleus and neuropil (referred to here as total aggregated mHTT) in the R6/1 mouse brain (Figure 10A). S830 reactivity was negligible in WT (percentage striatal FOV immunoreactive was 0.16%), indicating that it reflects a neuropathological marker in the R6/1 genotype. In R6/1s, the levels of mHTT varied across brain regions for both nuclear mHTT inclusions [F(Region)_5,102_ =17.808, p<0.001] and total mHTT aggregates [F(Region)_5,104_=86.98, p<0.001]. However, there was no influence of gender on either measure (nuclear mHTT: [F(Gender X Region)_5,102_=0.675, ns]; total mHTT: [F(Gender X Region)_5,104_=0.503, ns]). Indeed, a post-hoc analysis revealed that there was no gender-dependent difference between either the nuclear inclusions (Figure 10B) or total aggregated mHTT (Figure 10C) levels in the striatum, cortex or hippocampal subfields (dentate gyrus, CA1, CA2 or CA3). Generally, mHTT levels were positively correlated across all brain regions (Table S7). When considering male and female R6/1s together, total levels of mHTT were highly correlated across the CA1, CA2 and CA3 hippocampal subfields (r>0.753, p<0.0008), indicating a more ubiquitous detection in these areas. Interestingly, on TBM analysis of MRI, the hippocampus exhibited little atrophy despite a heavy molecular pathology.

**Figure 11:**
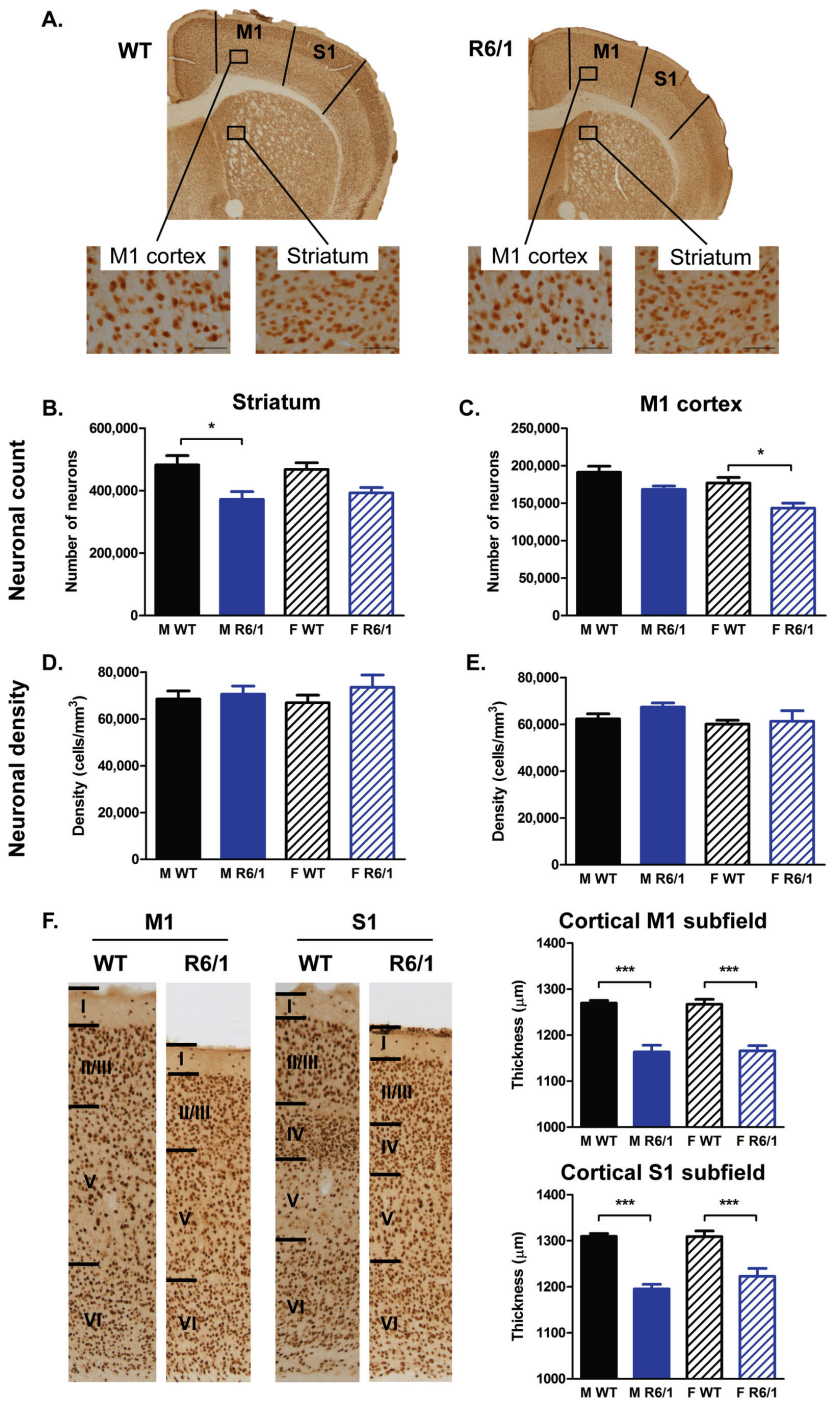
Histological analyses of NeuN-stained sections. (A) Sample images of 19 week old WT and R6/1 coronal sections stained for NeuN; scale bar 50 μm. There was a lower number of neurons in the striatum (B) and M1 cortex (C) in R6/1 mice, but no difference in neuronal density in these two regions (D & E). There was substantial cortical thinning (F) in both the M1 and S1 cortical subfields in R6/1 mice. All data analyzed using a two-way ANOVA (Genotype X Gender). Striatal measures of stereology: WT male n=10, R6/1 male n=9, WT female n=10, R6/1 female n=9; M1 cortical measures of stereology: WT male n=10, R6/1 male n=8, WT female n=10, R6/1 female n=9; cortical thickness in M1: WT male n=9, R6/1 male n=8, WT female n=10, R6/1 female n=9, and S1: WT male n=9, R6/1 male n=9, WT female n=9, R6/1 female n=9. All data presented as means ± SEM; *p<0.05, ***p<0.001.

 A stereological analysis of sections, stained for the neuronal marker NeuN (Figure 11A), revealed lower neuronal numbers in R6/1 compared to WTs, evident in both the striatum ([F(Genotype)_1,37_=14.546, p=0.001]; Figure 11B) and M1 cortex ([F(Genotype)_1,36_=15.432, p<0.001]; Figure 11C). There was no influence of gender on this drop in R6/1 neuronal number in either region (striatum: [F(Genotype x Gender)_1,37_=0.535, ns]; M1 cortex: [F(Genotype X Gender)_1,36_=0.55, ns]). The substantially smaller regional volumes in the R6/1s (striatum by 76.35% of WTs, M1 cortex by 81.35%) accompanied by this drop in neuronal numbers (striatum by 80.49% of WTs, M1 cortex by 84.29%) resulted in an absence of change in neuronal density in either region (Figure 11D&E; striatum: [F(Genotype)_1,37_=1.205, ns]; M1 cortex: [F(Genotype)_1,36_=0.837, ns]). Changes in neuronal number and volume were accompanied by substantial cortical thinning (Figure 11F) in both the M1 cortex [F(Genotype)_1,35_=92.105, p<0.001] and primary somatosensory (S1) cortex [F(Genotype)_1,35_=68.558, p<0.001], but gender had no influence on these measures (M1 cortex: [F(Genotype X Gender)_1,35_=0.55, ns]; S1 cortex [F(Genotype X Gender)_1,35_=1.347, ns]). 

Generally, there was a tendency towards strong positive correlations between neuronal number and neuronal density in the striatum of both WTs (males r=0.949, p<0.0033; females r=0.876, p<0.0033) and R6/1s (males r=0.927; p<0.0033, females r=0.825, ns), but not in the M1 cortex (Table S8). Interestingly there was no significant correlation between neuronal numbers in the striatum and M1 cortex for R6/1s (males r=0.079, ns; females r=0.638, ns) indicating that changes in neuronal number of R6/1 mice may not occur ubiquitously across the brain.

### The histopathological basis of brain abnormalities and behavioral impairments

By 17 weeks of age, R6/1 mice exhibited substantial brain atrophy visualized through MRI. By combining this imaging along with measures of histopathology, it is possible to investigate potential mechanisms behind this apparent brain volume loss. Nevertheless, there were no detectable associations between mHTT load and MRI-based regional brain changes (Table S9). Similarly, when considering the relationship between NeuN-based stereological data and MRI measures of brain abnormalities (Table S10), there were no correlations for R6/1 mice. Importantly, there was no correlation between neuronal numbers in either the striatum or M1 cortex with any MR measures of pathology, which may suggest that other, non-neuronal histopathological changes in the brain could have a greater or more direct influence on atrophy. Nevertheless, when considering male WT and R6/1 mice together, striatal volume determined through stereology strongly correlated with most MR-volumetry (striatum r=0.727, p<0.0033; cortex r=0.752, p<0.0033; hippocampus r=0.794, p<0.0033), indicating that indeed these two outcome measures were probing the same anatomical changes.

 Through combining assessments of behavioral performance with histopathological abnormalities, potential neurobiological mechanisms underlying functional deficits in R6/1 mice can be examined. Levels of mHTT in the brain, however, did not correlate with age-matched behavioral performance (Table S11), with the exception of a higher mHTT inclusion level in the hippocampal CA2 being associated with greater impairments in the swimming T-maze “cue-reversal” learning (r=0.925, p<0.0017). Similarly, behavioral performance did not show any association with stereological measures of pathology in the R6/1s (Table S12), with the exception of a smaller striatum being associated with an impaired learning at the cue learning phase of the swimming T-maze (r=-0.652, p<0.0033). Taken together, despite a few correlations of histological measures with single behavioral findings, there was an absence of direct correlative associations between these two outcome measures. It is therefore possible that a more complex relationship exists between the HD-like neuropathology and subsequent behavioral impairments in these mice.

## Discussion

Establishing how molecular pathology in Huntington’s disease (HD) leads to structural brain changes and the emergence of functional abnormalities remains a major challenge, both clinically in HD sufferers, and preclinically in mouse models of the disease. Moreover, for HD animals to be effective models in discovering novel therapeutic strategies, we need an understanding of how the disease-like phenotypes develop and relate to one another.

 Using an interdisciplinary approach, we have provided a detailed description of the progressive behavioral phenotype, brain atrophy and neuropathological abnormalities, akin to those observed in HD, in the transgenic R6/1 mouse model. The key findings were 1) R6/1 mice exhibited age-related deficits in both motor and cognitive behaviors detectable by 11 weeks of age; 2) MRI revealed the first changes in the R6/1 brain preceding behavioral dysfunction, as there was a smaller whole brain volume already at 9 weeks of age, although atrophy in various brain regions was only apparent later at 15 weeks; 3) accompanying these behavioral and brain abnormalities were typical HD histopathological features, including mHTT accumulation and neuronal loss; 4) there was, however, little evidence that there is a strong association between these typical histopathological markers of HD and behavioral deficits suggesting that other neurobiological changes might play an important role in the development of the impairments observed in HD.

### R6/1 mice mimic key clinical HD signs

Given the multi-faceted nature of clinical HD phenotypes, it is essential to thoroughly characterize the different animal models aiming to replicate the progressive pathology, as well as the behavioral phenotype of HD. To characterize behavioral changes in the R6/1 mouse model of HD, a battery of tests aimed at probing motor and cognitive capabilities was applied to investigate the emergence of deficits across different functions.

 The earliest detectable abnormality was a motor coordination impairment on the rotarod at 11 weeks of age, although it is important to note that this was the earliest age tested and it is therefore possible that this deficit even precedes this age. This early impairment is consistent with a deficit detected in a previous study even earlier, at 2 months of age [20]. This motor coordination task is therefore a useful marker for early HD-like phenotype detection, detectable even before overt body weight loss. Interestingly, this task was found to be impaired in the R6/2 at a similar age, developing around 9 and 13 weeks [19]. We also noted robust decreases in grip strength, which developed between 10 and 19 weeks of age. To our knowledge, this is the first description of the progressive development of grip strength deficits in this mouse line, although it has been well described in R6/2 mice [49,50,51]. These impairments reflect the decline of neuromuscular function and muscle strength, common features of HD. General activity in an open-field, however, was unaltered in R6/1 mice. These data are consistent with those showing an absence of altered R6/1 activity measures over 30 min up to 14 week of age [52]. Yet, others reported diminished exploratory activity as a late (23 weeks, [53]), as well as an early phenomenon (6 weeks, [54]) in R6/1 mice. Longer (24h) assessments of locomotor activity revealed also evidence of hypoactivity from 18 weeks of age [55]. Comparing activity assessments in R6/1s from various literatures is nevertheless difficult given the gross inconsistencies of testing conditions.

In addition to motor impairments, R6/1 mice developed deficits in visuo-spatial cue-based learning, and its subsequent reversal, at 15 weeks of age. The neuroanatomical mechanisms underlying these two distinct forms of learning are complex, but the reversal of cued learning requires a higher degree of cognitive flexibility. Consequently, this is more cognitively demanding, as reflected by the length of time taken to acquire the new rule. Orbital and prefrontal cortex [56], as well as frontostriatal brain regions [57] have been implicated in variants of reversal learning in mice. Abnormalities in the neuronal functioning within these regions have been stipulated to be involved in reversal learning deficits in several HD mouse models, including the R6/2 line [58,59,60]. Consistent with these observations, we found that smaller striatal volumes were associated with cue-reversal learning deficits in female R6/1s.

### Neuropathological correlates of behavior

As expected, we detected the extensive accumulation of mHTT in the R6/1 mouse brain. As in the R6/2 mouse line [19], there were no differences in protein abundance between male and female R6/1 mice. It is worthy of note that the relative amount of mHTT in the R6/1 brain regions investigated is comparable to those in the R6/2. In the current study, we quantified mHTT levels at 19 weeks of age only, however, a previous characterization has demonstrated mHTT presence in the R6/1 brain from as early as 2 months, which greatly increased by 4 months [21].

The R6/1 and R6/2 lines had other similarities in brain pathology expression, as both models exhibited a reduction in brain volumes over time, detected here using longitudinal *in vivo* MRI. As with the R6/2 mice [19], the R6/1 striatum appears not to shrink over time, but rather does not grow at the same rate as WTs. A similar effect of this absence of normal striatal growth in the R6/1, rather than regional shrinkage, has been detected previously through histological methods [21]. Interestingly, unlike the striatal volume that remains relatively stable from 9 to 17 weeks (only a 4.19 % drop), the cortical volumes appear to shrink more significantly over time (13.24% drop). Again, these patterns of regional brain volume change are consistent with those described in the R6/2 mice previously [19]. These regional patterns of brain changes are distinct from those generally observed in clinical investigations of longitudinal brain atrophy in HD. MRI has detected progressive atrophy of both the basal ganglia and neocortex [61], but others have shown progressive atrophy of the striatum in patients with >10 years of HD onset, and this progresses with disease [62,63], although general grey-matter changes could not be detected as early [62]. Indeed, striatal volume loss was a good predictor of disease diagnosis in a separate study [64]. Therefore, unlike our recent data from mouse models, the evidence from clinical studies of HD indicate a robust, early change in striatal volume in HD sufferers which continues to develop as the disease progresses.

Alongside manually segmented ROI volumetry studies, TBM allowed an unbiased, sensitive, assessment of volume change across the whole brain, including sub-regional changes [65,66]. Interestingly, TBM revealed a more substantial pathology in the male R6/1 mice compared to females, a phenomenon that was less easy to resolve through manual segmentation. Collectively, both manually defined MR volumetry and TBM demonstrated age-related loss of grey matter, whilst sparing white matter in the R6/1 mice. However, TBM allowed for more precise detection of shrinkage of anatomical loci within larger brain regions, such as retrosplenial areas alongside a relatively spared anterior cingulate within the cortex. Interestingly, although there was a general widespread atrophy occurring across the whole brain in R6/1, the hippocampus was generally spared, despite this region being heavily impacted by mHTT. 

 Despite various similarities with R6/2 mice, there were also several key differences in the pathological progression of R6/1 mice. One major difference between the brain pathology of the R6/1 and R6/2 mice is the presence of neuronal loss in R6/1s. Previously, we have detected no difference in neuronal number in R6/2 mice at 14 weeks of age [19], however, we detected neuronal loss in the brain of R6/1 mice at 19 weeks of age, consistent with a previous study which also described neuronal loss in R6/1 [21]. There were no correlations between neuronal loss and brain region volume change in the R6/1 mice, which may indicate that non-neuronal changes are responsible for the gross brain volume change in the R6/1. Such changes may include loss of microglia and extracellular matrix molecule, both being features of R6/2 mice [67,68]. Neuronal density was highly elevated in R6/2 mice [19], which was likely a result of a lack of neuronal loss coupled with restricted striatal growth. Conversely, in the current study R6/1 mice exhibit both neuronal loss and volume differences compared to WT mice, thus resulting in a normal cellular density. Heightened cellular density in the R6/2 mice may have resulted in lowered tissue water content, which would, as a result, shorten T2 relaxation values. It is therefore conceivable that the absence of cellular density change in the R6/1s, results in normal tissue water content levels and hence, the generally unaffected tissue T2 relaxation values described here.

To determine whether there were clear neuropathological substrates responsible for the robust behavioral dysfunction, a correlative analysis was performed between these two outcome measures. There were few associations between brain abnormalities determined through MRI and age-matched behavioral performance, although both progressively worsened with age in the R6/1. Studies have revealed that abnormal neuronal functioning underlies behavioral dysfunction in the R6/2 mouse line [69,70], and a disrupted neuronal activity has also been reported as in R6/1 mice [71]. It is therefore possible that abnormalities in neuronal activity, rather than brain tissue loss, underlie the functional phenotypes in R6 mice. 

### Intensive behavioral testing may be detrimental to longitudinal studies

R6/1 mice typically survive until around 40 weeks of age [5], and can be behaviorally characterized up to 7 months of age [20]. The current investigation took the R6/1 mice to 18 weeks of age, whereby they reached a predetermined humane endpoint approximately 12 weeks earlier than anticipated. The experiment was consequently stopped. Given the nature of the intense behavioral characterization undergone here, as well as the repeated anesthesia for MRI, this may be in part responsible for an anticipation of the humane endpoint. It is hence important to consider the here presented results in this context.

 As detailed in the experimental design, mice were subjected first to repeated swimming exposure followed by an intense four weeks of behavioral testing. Alongside our test animals, a separate cohort of WT and R6/1 mice were aged for tissue collection. Body weight, a common, albeit superficial measure of animal welfare, was lower in the animals exposed to the intense behavioral exposure for both WTs (10.85% lower) and R6/1s (13.58% lower). The increase in general activity and exercise during behavioral testing is likely to reduce body weight at the same caloric intake.

 Unexpected and uncontrollable stressors have historically been used to model many functional deficits associated with depression in laboratory animals [72] and standard paradigms aimed at probing behavior in animals can themselves induce states of anxiety and emotional stress [73]. Indeed, elevated levels of the stress-response hormone, corticosterone, interferes with normal cognitive functioning [74]. This is of particular concern when studying animal models of brain disorders given the well described interactions between emotional stressors and neurodegenerative disease [75]. In fact, it is possible that transgenic models of HD are more susceptible to stress-related insults given a hyperactivity of the stress-response pathway, the hypothalamic-pituitary-adrenal (HPA) axis, as has been reported in both R6/2 [76] and R6/1 [77] mice. The extent, intensity and duration of behavioral characterization of R6 mice (regardless of any possible heightened susceptibility to stress-related insults) should therefore be carefully considered, as there is a risk of modifying disease progression.

## Conclusions

This study demonstrated both motor and cognitive deficits in the R6/1 mouse line with concomitant brain atrophy as determined through MRI. Accumulation of mHTT and a loss of neurons in key brain structures was also evident post-mortem, indicating that neurobiological changes underlie changes in brain structure and behavior. However, there was little evidence of a direct link between brain pathology and behavioral deficits. The R6/1 mouse line therefore replicates several key behavioral and neuropathological features of HD, but presently no mechanistic link between these could be established. Investigation of the early stages of behavioral impairments in this model combined with a more extensive histopathological and molecular analysis might be required to establish specific associations that link molecular pathology to a specific behavioral deficit.

## Supporting Information

Figure S1 **Striatal and M1 cortical inclusion criteria for stereology.** Sample coronal brain sections stained with NeuN with guides of neuroanatomical boundaries for inclusion of the striatum and M1 cortex for stereological analysis.
(TIF)

Table S1 **Number of animals used.** All tests were conducted on the same cohort of animals. However, there was a variable subject number for each test due to either death during the study, to the occasional missing data sample or to the exclusion of statistical outliers. RR = rotarod, LMA = locomotor activity in an open field, GS FL = grip strength of the fore-limbs, GS 4L = grip strength of the fore- and hind limbs, TM CL = swimming T-maze cue learning, TM CR = swimming T-maze cue reversal, FC CS = fear conditioning cue recall (total immobility over 25 cue exposures), FC CT = fear conditioning contextual recall, OD = odor discrimination, SI = social interaction, STR = striatum, CTX = cortex, HIPP = hippocampus, CC = corpus callosum, WB = whole brain, MUSC = muscle tissue, DG = dentate gyrus, CA1 = hippocampal CA1 subfield, CA2 = hippocampal CA2 subfield, CA3 = hippocampal CA3 subfield, M1 CTX = M1 cortex, S1 CTX = S1 cortex.
(PDF)

Table S2 **Main effects derived from statistical analyses.** Main effects derived from two-way and three-way ANOVAs. LMA = locomotor activity in an open field, GS FL = grip strength of the forelimbs, GS 4L = grip strength of the fore- and hind limbs, TM CL = swimming T-maze cue learning, TM CR = swimming T-maze cue reversal, RR = rotarod, OD = odor discrimination, SI = social interaction, FC CS = fear conditioning cue recall (total immobility over 25 cue exposures), FC CT = fear conditioning contextual recall, STR = striatum, CTX = cortex, HIPP = hippocampus, CC = corpus callosum, WB = whole brain, MUSC = muscle tissue, DG = dentate gyrus, CA1 = hippocampal CA1 subfield, CA2 = hippocampal CA2 subfield, CA3 = hippocampal CA3 subfield, M1 CTX = M1 cortex, S1 CTX = S1 cortex.
(PDF)

Table S3 **Correlations of behavioral measures taken between 6 and 12 weeks.** Correlations of performance at behavioral tasks tested between 6 and 12 weeks, presented as Pearson r values. RR = rotarod, LMA = locomotor activity in an open field, GS FL = grip strength of the forelimbs, GS 4L = grip strength of the fore- and hind limbs, TM CL = swimming T-maze cue learning, TM CR = swimming T-maze cue reversal, FC CS = fear conditioning cue recall (total immobility over 25 cue exposures), FC CT = fear conditioning contextual recall, OD = odor discrimination, SI = social interaction.
(PDF)

Table S4 **Correlations of behavioral measures taken between 15 and 19 weeks.** Correlations of performance at behavioral tasks tested between 15 and 19 weeks, presented as Pearson r values. LMA = locomotor activity in an open field, GS FL = grip strength of the forelimbs, GS 4L = grip strength of the fore- and hind limbs, TM CL = swimming T-maze cue learning, TM CR = swimming T-maze cue reversal. *Statistically significant after Bonferroni Correction (adjusted p value 0.005).
(PDF)

Table S5 **Correlations of MRI measures.** Correlations of measures of pathological burden assessed through MRI across time, presented as Pearson r values. STR = striatum, CTX = cortex, HIPP = hippocampus, CC = corpus callosum, WB = whole brain, MUSC = muscle tissue. *Statistically significant after Bonferroni Correction (adjusted p value 0.0011).
(PDF)

Table S6 **Correlations of behavioral abnormalities against brain abnormalities determined through MRI.** Correlations of behavioral data against age-matched MRI measures taken between either 6 and 12 weeks (matched to 9 week MRI data) and 15 and 19 weeks (matched to 17 week MRI data), presented as Pearson r values. RR = rotarod, LMA = locomotor activity in an open field, GS FL = grip strength of the forelimbs, GS 4L = grip strength of the fore- and hind limbs, TM CL = swimming T-maze cue learning, TM CR = swimming T-maze cue reversal, FC CS = fear conditioning cue recall (total immobility over 25 cue exposures), FC CT = fear conditioning contextual recall, OD = odor discrimination, SI = social interaction, STR = striatum, CTX = cortex, HIPP = hippocampus, CC = corpus callosum, WB = whole brain, MUSC = muscle tissue. *Statistically significant after Bonferroni Correction (adjusted p value 0.001).
(PDF)

Table S7 **Correlations of mHTT abundance across different brain regions.** Correlations of total detectable mHTT (Total aggregated mHTT) and nuclear inclusions (Nuc mHTT) across the six brain regions investigated in 19 week old R6/1 mice only, presented as Pearson r values. STR = striatum, CTX = cortex, DG = dentate gyrus, CA1 = hippocampal CA1 subfield, CA2 = hippocampal CA2 subfield, CA3 = hippocampal CA3 subfield. *Statistically significant after Bonferroni Correction (adjusted p value 0.0008).
(PDF)

Table S8 **Correlations of neuronal characteristics.** Correlations of neuronal number (Neur no.), density (Neur dens.) and regional volume determined through stereological analyses of NeuN-strained brain sections in 19 week old mice, presented as Pearson r values. STR = striatum, M1 CTX = M1 cortex. *Statistically significant after Bonferroni Correction (adjusted p value 0.0033).
(PDF)

Table S9 **Correlations of mHTT levels versus MRI measures of brain abnormalities.** Correlations of both post-mortem (at 19 weeks) total aggregated mHTT levels and nuclear inclusions (Nuc mHTT) against measures of brain pathology through MRI at 17 weeks, presented as Pearson r values. STR = striatum, CTX = cortex, DG = dentate gyrus, CA1 = hippocampal CA1 subfield, CA2 = hippocampal CA2 subfield, CA3 = hippocampal CA3 subfield, HIPP = hippocampus, CC = corpus callosum, WB = whole brain, MUSC = muscle tissue.
(PDF)

Table S10 **Correlations of neuronal characteristics versus MRI measures of brain abnormalities.** Correlations of post-mortem neuronal number (Neur no.), density (Neur dens.) and regional volume determined through stereology on NeuN-stained brain sections against measures of brain pathology through MRI at 17 weeks of age, presented as Pearson r values. STR = striatum, M1 CTX = primary motor cortex, CTX = cortex, HIPP = hippocampus, CC = corpus callosum, WB = whole brain, MUSC = muscle tissue. *Statistically significant after Bonferroni Correction (adjusted p value 0.0033).
(PDF)

Table S11 **Correlations of mHTT levels versus behavioral performance.** Correlations of post-mortem (19 weeks) total mHTT and nuclear inclusions (Nuc mHTT) against behavioral phenotypes exhibited by R6/1 mice between 15 and 19 weeks of age, presented as Pearson r values. LMA = locomotor activity in an open field, GS FL = grip strength of the forelimbs, GS 4L = grip strength of the fore- and hind limbs, TM CL = swimming T-maze cue learning, TM CR = swimming T-maze cue reversal, STR = striatum, CTX = cortex, DG = dentate gyrus, CA1 = hippocampal CA1 subfield, CA2 = hippocampal CA2 subfield, CA3 = hippocampal CA3 subfield. *Statistically significant after Bonferroni Correction (adjusted p value 0.0017).
(PDF)

Table S12 **Correlations of neuronal characteristics versus behavioral performance.** Correlations of post-mortem (19 weeks) neuronal number (Neur no.), density (Neur dens.) and regional volume determined through stereology on NeuN-stained brain sections against behavioral performance recorded between 15 and 19 weeks of age, presented as Pearson r values. LMA = locomotor activity in an open field, GS FL = grip strength of the forelimbs, GS 4L = grip strength of the fore- and hind limbs, TM CL = swimming T-maze cue learning, TM CR = swimming T-maze cue reversal, STR = striatum, M1 CTX = primary motor cortex. *Statistically significant after Bonferroni Correction (adjusted p value 0.0033).
(PDF)
