# Supplementary figures and images for: Correlations of Behavioral Deficits with Brain Pathology Assessed through Longitudinal MRI and Histopathology in the R6/1 Mouse Model of Huntington’s Disease
Source: PLoS One. 2013 Dec 19;8(12):e84726. doi: 10.1371/journal.pone.0084726 (PMC3868608; doi:10.1371/journal.pone.0084726)

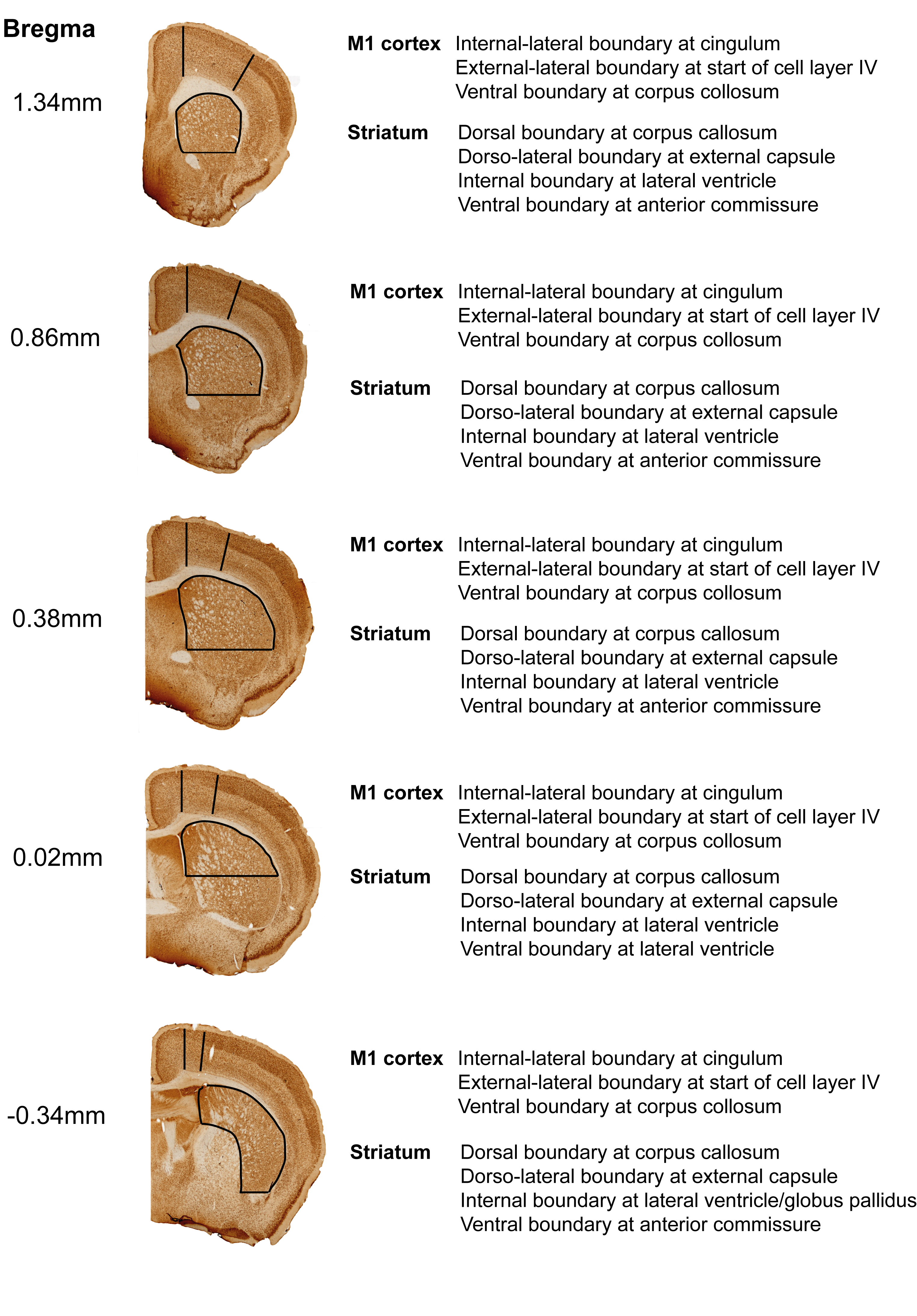

Supplement: Figure S1 — Striatal and M1 cortical inclusion criteria for stereology. Sample coronal brain sections stained with NeuN with guides of neuroanatomical boundaries for inclusion of the striatum and M1 cortex for stereological analysis. (TIF) [file pone.0084726.s001.tif]
