# Supplementary material for: Correlations of Behavioral Deficits with Brain Pathology Assessed through Longitudinal MRI and Histopathology in the R6/1 Mouse Model of Huntington’s Disease
Source: PLoS One. 2013 Dec 19;8(12):e84726. doi: 10.1371/journal.pone.0084726 (PMC3868608; doi:10.1371/journal.pone.0084726)
Supplement: Table S1 — Number of animals used. All tests were conducted on the same cohort of animals. However, there was a variable subject number for each test due to either death during the study, to the occasional missing data sample or to the exclusion of statistical outliers. RR = rotarod, LMA = locomotor activity in an open field, GS FL = grip strength of the fore-limbs, GS 4L = grip strength of the fore- and hind limbs, TM CL = swimming T-maze cue learning, TM CR = swimming T-maze cue reversal, FC CS = fear conditioning cue recall (total immobility over 25 cue exposures), FC CT = fear conditioning contextual recall, OD = odor discrimination, SI = social interaction, STR = striatum, CTX = cortex, HIPP = hippocampus, CC = corpus callosum, WB = whole brain, MUSC = muscle tissue, DG = dentate gyrus, CA1 = hippocampal CA1 subfield, CA2 = hippocampal CA2 subfield, CA3 = hippocampal CA3 subfield, M1 CTX = M1 cortex, S1 CTX = S1 cortex. (PDF) [file pone.0084726.s002.pdf]

	Body weight 6w	10w	14w	18w	19w	Body temperature 6w	10w	14w	18w
**Male WT**	11	11	11	11	11	11	11	11	11
**Male R61**	9	9	9	9	9	9	9	9	9
**Female WT**	10	10	10	10	10	10	10	10	10
**Female R61**	10	10	10	8	8	10	10	10	8

	RR 11w	LMA 10w	19w	GS FL 10w	19w	GS 4L 10w	19w
**Male WT**	11	11	11	11	11	11	11
**Male R61**	9	9	9	9	9	9	9
**Female WT**	10	10	10	10	10	10	10
**Female R61**	10	9	8	10	8	10	8

	TM CL 6w	15w	TM CR 6w	15w	FC CS 12w	FC CT 12w	OD 10w	SI 10w
**Male WT**	10	11	10	10	11	11	11	10
**Male R61**	9	9	9	8	9	9	8	9
**Female WT**	10	10	10	10	7	8	9	10
**Female R61**	10	9	10	9	8	7	8	10

	STR 9w	17w	CTX 9w	17w	HIPP 9w	17w	CC 9w	17w	WB 9w	17w
**Male WT**	11	11	11	11	11	11	11	11	11	11
**Male R61**	9	9	9	9	9	9	9	9	9	9
**Female WT**	10	10	10	10	10	10	10	10	10	10
**Female R61**	10	9	10	9	10	9	10	9	10	9

	STR 9w	17w	CTX 9w	17w	HIPP 9w	17w	CC 9w	17w	MUSC 9w	17w
**Male WT**	11	11	11	11	11	11	11	11	10	11
**Male R61**	9	9	9	9	9	9	9	9	9	9
**Female WT**	10	9	8	10	10	9	10	9	10	10
**Female R61**	10	9	10	9	10	9	10	8	10	9

	Total mHTT STR	CTX	DG	CA1	CA2	CA3	Nuclear inclusions STR	CTX	DG	CA1	CA2	CA3
**Male R61**	9	9	9	9	8	9	9	9	9	8	9	9
**Female R61**	9	8	8	9	9	9	9	9	8	8	7	9

	STR Nu No	Nu Dn	Vol	M1 CTX Nu No	Nu Dn	Vol	CTX THICK M1	S1
**Male WT**	10	10	10	10	10	10	9	9
**Male R61**	9	9	9	8	8	8	8	9
**Female WT**	10	10	10	10	10	10	10	9
**Female R61**	9	9	9	9	9	9	9	9

Animal(s) lost due to death
Animal(s) lost due to data remova/technical error
Animal(s) lost due to both death and data removal/technical error
