# Supplementary material for: Correlations of Behavioral Deficits with Brain Pathology Assessed through Longitudinal MRI and Histopathology in the R6/1 Mouse Model of Huntington’s Disease
Source: PLoS One. 2013 Dec 19;8(12):e84726. doi: 10.1371/journal.pone.0084726 (PMC3868608; doi:10.1371/journal.pone.0084726)
Supplement: Table S2 — Main effects derived from statistical analyses. Main effects derived from two-way and three-way ANOVAs. LMA = locomotor activity in an open field, GS FL = grip strength of the forelimbs, GS 4L = grip strength of the fore- and hind limbs, TM CL = swimming T-maze cue learning, TM CR = swimming T-maze cue reversal, RR = rotarod, OD = odor discrimination, SI = social interaction, FC CS = fear conditioning cue recall (total immobility over 25 cue exposures), FC CT = fear conditioning contextual recall, STR = striatum, CTX = cortex, HIPP = hippocampus, CC = corpus callosum, WB = whole brain, MUSC = muscle tissue, DG = dentate gyrus, CA1 = hippocampal CA1 subfield, CA2 = hippocampal CA2 subfield, CA3 = hippocampal CA3 subfield, M1 CTX = M1 cortex, S1 CTX = S1 cortex. (PDF) [file pone.0084726.s003.pdf]

Physiological measures

	Body Weight		Body temp	
	F	Sig.	F	Sig.
G'type	$16.786_{(1,195)}$	p<0.001	$5.768_{(1,157)}$	p=0.018
Gender	$175.645_{(1,195)}$	p<0.001	$8.152_{(1,157)}$	p=0.005
Age	$35.056_{(4,195)}$	p<0.001	$4.678_{(3,157)}$	p=0.004
G'type X Gender	$0.845_{(1,195)}$	p=0.359	$0.18_{(1,157)}$	p=0.672
G'type X Age	$6.173_{(4,195)}$	p<0.001	$0.293_{(3,157)}$	p=0.83
Gender X Age	$1.629_{(4,195)}$	p=0.169	$0.574_{(3,157)}$	p=0.633
G'type X Gender X Age	$0.605_{(4,195)}$	p=0.659	$0.839_{(3,157)}$	p=0.475

Lognitudinal behaviors

	LMA		GS FL		GS 4L		TM CL		TM CR	
	F	Sig.	F	Sig.	F	Sig.	F	Sig.	F	Sig.
G'type	$7.296_{(1,76)}$	p=0.009	$6.588_{(1,77)}$	p=0.012	$17.888_{(1,77)}$	p<0.001	$5.066_{(1,77)}$	p=0.028	$3.246_{(1,75)}$	p=0.076
Gender	$1.378_{(1,76)}$	p=0.244	$2.651_{(1,77)}$	p=0.108	$15.784_{(1,77)}$	p<0.001	$1.336_{(1,77)}$	p=0.252	$0.206_{(1,75)}$	p=0.651
Age	$9.201_{(1,76)}$	p=0.003	$3.248_{(1,77)}$	p=0.076	$0.31_{(1,77)}$	p=0.861	$18.395_{(1,77)}$	p<0.001	$0.002_{(1,75)}$	p=0.964
G'type X Gender	$2.115_{(1,76)}$	p=0.15	$0.1_{(1,77)}$	p=0.752	$1.033_{(1,77)}$	p=0.313	$0.185_{(1,77)}$	p=0.668	$0.348_{(1,75)}$	p=0.557
G'type X Age	$0.443_{(1,76)}$	p=0.508	$6.721_{(1,77)}$	p=0.012	$13.107_{(1,77)}$	p=0.001	$2.663_{(1,77)}$	p=0.107	$19.17_{(1,75)}$	p<0.001
Gender X Age	$0.75_{(1,76)}$	p=0.784	$3.523_{(1,77)}$	p=0.065	$1.913_{(1,77)}$	p=0.171	$0.043_{(1,77)}$	p=0.837	$0.48_{(1,75)}$	p=0.828
G'type X Gender X Age	$0.519_{(1,76)}$	p=0.474	$0.256_{(1,77)}$	p=0.614	$2.093_{(1,77)}$	p=0.152	$6.496_{(1,77)}$	p=0.013	$0.735_{(1,75)}$	p=0.394

Behavior taken at a single time point

	RR		OD		SI		FC CR		FC CT	
	F	Sig	F	Sig	F	Sig	F	Sig	F	Sig
G'type	$20.794_{(1,39)}$	p<0.001	$0.349_{(1,35)}$	p=0.559	$0.253_{(1,38)}$	p=0.618	$5.59_{(1,34)}$	p=0.025	$6.006_{(1,34)}$	p=0.02
Gender	$4.062_{(1,39)}$	p=0.051	$0.204_{(1,35)}$	p=0.654	$0.704_{(1,38)}$	p=0.407	$0.785_{(1,34)}$	p=0.383	$0.156_{(1,34)}$	p=0.696
Gender X G'type	$3.918_{(1,39)}$	p=0.055	$0.046_{(1,35)}$	p=0.832	$0.626_{(1,38)}$	p=0.434	$0.218_{(1,34)}$	p=0.644	$0.004_{(1,34)}$	p=0.951

MRI - volumetry

	STR		CTX		HIPP		CC		WB	
	F	Sig	F	Sig	F	Sig	F	Sig	F	Sig
G'type	$26.481_{(1,78)}$	p<0.001	$61.667_{(1,78)}$	p<0.001	$64.687_{(1,78)}$	p<0.001	$2.819_{(1,78)}$	p=0.098	$79.172_{(1,78)}$	p<0.001
Gender	$0.175_{(1,78)}$	p=0.677	$0.367_{(1,78)}$	p=0.546	$0.172_{(1,78)}$	p=0.68	$0.036_{(1,78)}$	p=0.851	$0.613_{(1,78)}$	p=0.436
Age	$0.133_{(1,78)}$	p=0.716	$41.62_{(1,78)}$	p<0.001	$2.667_{(1,78)}$	p=0.107	$2.871_{(1,78)}$	p=0.095	$2.694_{(1,78)}$	p=0.105
G'type X Gender	$0.464_{(1,78)}$	p=0.498	$0.033_{(1,78)}$	p=0.856	$0.056_{(1,78)}$	p=0.814	$4.032_{(1,78)}$	p=0.048	$0.138_{(1,78)}$	p=0.711
G'type X Age	$3.019_{(1,78)}$	p=0.087	$5.224_{(1,78)}$	p=0.025	$11.447_{(1,78)}$	p=0.001	$1.519_{(1,78)}$	p=0.222	$7.501_{(1,78)}$	p=0.008
Gender X Age	$1.291_{(1,78)}$	p=0.26	$1.214_{(1,78)}$	p=0.274	$0.099_{(1,78)}$	p=0.755	$2.072_{(1,78)}$	p=0.154	$1.562_{(1,78)}$	p=0.215
G'type X Gender X Age	$0.897_{(1,78)}$	p=0.347	$0.016_{(1,78)}$	p=0.898	$0.055_{(1,78)}$	p=0.815	$5.002_{(1,78)}$	p=0.028	$0.01_{(1,78)}$	p=0.92

MRI - T2 relaxivity

	STR		CTX		HIPP		CC		MUSC	
	F	Sig	F	Sig	F	Sig	F	Sig	F	Sig
G'type	$13.294_{(1,77)}$	p=0.001	$1.569_{(1,76)}$	p=0.215	$0.519_{(1,77)}$	p=0.474	$4.403_{(1,76)}$	p=0.04	$0.023_{(1,77)}$	p=0.88
Gender	$1.508_{(1,77)}$	p=0.224	$0.186_{(1,76)}$	p=0.668	$0.798_{(1,77)}$	p=0.375	$0.138_{(1,76)}$	p=0.711	$6.035_{(1,77)}$	p=0.017
Age	$9.459_{(1,77)}$	p=0.003	$25.547_{(1,76)}$	p<0.001	$14.54_{(1,77)}$	p<0.001	$0.22_{(1,76)}$	p=0.641	$0.378_{(1,77)}$	p=0.541
G'type X Gender	$2.74_{(1,77)}$	p=0.102	$1.346_{(1,76)}$	p=0.25	$3.916_{(1,77)}$	p=0.052	$0.314_{(1,76)}$	p=0.577	$0.362_{(1,77)}$	p=0.55
G'type X Age	$0.017_{(1,77)}$	p=0.897	$1.081_{(1,76)}$	p=0.302	$2.256_{(1,77)}$	p=0.138	$0.094_{(1,76)}$	p=0.76	$2.253_{(1,77)}$	p=0.138
Gender X Age	$0.017_{(1,77)}$	p=0.898	$0.003_{(1,76)}$	p=0.955	$0.138_{(1,77)}$	p=0.711	$0.749_{(1,76)}$	p=0.39	$0.286_{(1,77)}$	p=0.594
G'type X Gender X Age	$1.662_{(1,77)}$	p=0.202	$3.194_{(1,76)}$	p=0.078	$3.499_{(1,77)}$	p=0.066	$2.507_{(1,76)}$	p=0.118	$1.714_{(1,77)}$	p=0.195

Stereological measures

	STR				M1 CTX				CTX THICKNESS			
	No.		Dens		No.		Dens.		M1		S1	
	F	Sig	F	Sig	F	Sig	F	Sig	F	Sig	F	Sig
G'type	$14.546_{(1,37)}$	p=0.001	$1.205_{(1,37)}$	p=0.28	$15.432_{(1,36)}$	p<0.001	$0.837_{(1,36)}$	p=0.367	$92.105_{(1,35)}$	p<0.001	$68.558_{(1,35)}$	p<0.001
Gender	$0.17_{(1,37)}$	p=0.898	$0.031_{(1,37)}$	p=0.861	$7.491_{(1,36)}$	p=0.01	$3.102_{(1,36)}$	p=0.087	$0.001_{(1,35)}$	p=0.978	$1.196_{(1,35)}$	p=0.282
Gender X G'type	$0.535_{(1,37)}$	p=0.469	$0.329_{(1,37)}$	p=0.57	$0.55_{(1,36)}$	p=0.463	$0.466_{(1,36)}$	p=0.5	$0.055_{(1,35)}$	p=0.816	$1.347_{(1,35)}$	p=0.254
