# Supplementary material for: Correlations of Behavioral Deficits with Brain Pathology Assessed through Longitudinal MRI and Histopathology in the R6/1 Mouse Model of Huntington’s Disease
Source: PLoS One. 2013 Dec 19;8(12):e84726. doi: 10.1371/journal.pone.0084726 (PMC3868608; doi:10.1371/journal.pone.0084726)
Supplement: Table S3 — Correlations of behavioral measures taken between 6 and 12 weeks. Correlations of performance at behavioral tasks tested between 6 and 12 weeks, presented as Pearson r values. RR = rotarod, LMA = locomotor activity in an open field, GS FL = grip strength of the forelimbs, GS 4L = grip strength of the fore- and hind limbs, TM CL = swimming T-maze cue learning, TM CR = swimming T-maze cue reversal, FC CS = fear conditioning cue recall (total immobility over 25 cue exposures), FC CT = fear conditioning contextual recall, OD = odor discrimination, SI = social interaction. (PDF) [file pone.0084726.s004.pdf]

		RR	LMA	GS FL	GS 4L	TM CL	TM CR	FC CS	FC CT	OD	SI	
**WT males**	**RR**		*0.337*	*-0.265*	*0.116*	*-0.058*	*0.033*	*0.456*	*-0.182*	*-0.537*	*-0.631*	***WT females***
	**LMA**	0.62		*-0.077*	*-0.332*	*-0.281*	*-0.506*	*0.745*	*-0.05*	*-0.14*	*-0.113*	
	**GS FL**	-0.014	0.078		*0.387*	*-0.676*	*0.127*	*0.4*	*-0.247*	*0.22*	*-0.275*	
	**GS 4L**	0.22	0.113	0.413		*-0.362*	*-0.116*	*0.003*	*0.207*	*-0.091*	*-0.446*	
	**TM CL**	-0.704	-0.531	0.345	-0.074		*0.379*	*-0.347*	*-0.293*	*-0.435*	*0.595*	
	**TM CR**	0.095	0.099	-0.199	-0.187	-0.396		*0.163*	*-0.643*	*-0.076*	*-0.018*	
	**FC CS**	-0.055	0.02	0.301	0.508	0.367	-0.521		*0.05*	*0.18*	*-0.637*	
	**FC CT**	0.364	-0.145	-0.292	0.179	-0.177	-0.004	0.448		*0.631*	*-0.196*	
	**OD**	0.462	0.123	0.065	0.425	0.011	-0.461	0.303	0.442		*-0.161*	
	**SI**	-0.348	-0.145	0.192	0.485	0.466	-0.748	0.555	-0.065	0.542		
**R6/1 males**	**RR**		*-0.523*	*-0.32*	*0.059*	*0.061*	*-0.143*	*0.031*	*-0.052*	*-0.61*	*0.195*	***R6/1 females***
	**LMA**	0.005		*0.135*	*-0.146*	*0.051*	*0.201*	*-0.689*	*-0.262*	*-0.147*	*0.034*	
	**GS FL**	-0.137	0.167		*0.829*	*-0.096*	*-0.185*	*0.169*	*0.05*	*-0.001*	*-0.122*	
	**GS 4L**	0.268	0.264	0.562		*0.111*	*-0.067*	*0.23*	*-0.252*	*0.116*	*-0.09*	
	**TM CL**	-0.195	0.741	-0.131	-0.165		*0.118*	*-0.238*	*-0.869*	*0.408*	*0.334*	
	**TM CR**	0.218	0.339	-0.073	0.602	-0.082		*-0.637*	*-0.618*	*-0.059*	*0.381*	
	**FC CS**	-0.081	-0.492	0.013	-0.287	-0.203	-0.455		*0.633*	*0.607*	*-0.134*	
	**FC CT**	0.005	0.43	-0.527	-0.14	0.352	0.187	0.029		*-0.211*	*-0.301*	
	**OD**	0.059	0.43	-0.365	-0.176	0.126	0.615	-0.229	0.516		*-0.176*	
	**SI**	-0.579	0.061	-0.261	-0.567	0.087	0.071	0.038	0.177	0.69		
**WT & R6/1 males**	**RR**		*0.362*	*-0.161*	*0.345*	*0.104*	*0.245*	*0.321*	*0.312*	*-0.441*	*-0.363*	***WT & R6/1 females***
	**LMA**	0.525		*0.025*	*-0.08*	*-0.044*	*-0.084*	*0.088*	*0.221*	*-0.191*	*-0.133*	
	**GS FL**	-0.061	0.095		*0.556*	*-0.243*	*-0.047*	*0.153*	*-0.095*	*0.129*	*-0.197*	
	**GS 4L**	0.108	0.058	0.482		*0.003*	*0.061*	*0.234*	*0.269*	*-0.058*	*-0.331*	
	**TM CL**	-0.585	-0.15	0.074	-0.039		*0.222*	*-0.138*	*-0.298*	*0.118*	*0.359*	
	**TM CR**	0.183	0.236	-0.137	0.179	-0.292		*-0.383*	*-0.414*	*-0.138*	*0.105*	
	**FC CS**	0.123	0.034	0.139	0.014	-0.166	-0.345		*0.521*	*0.355*	*-0.153*	
	**FC CT**	0.363	0.213	-0.373	-0.051	-0.125	0.12	0.396		*0.186*	*-0.193*	
	**OD**	0.268	0.179	-0.13	0.146	0.08	-0.029	0.038	0.363		*-0.173*	
	**SI**	-0.144	0.162	-0.075	-0.282	-0.028	-0.179	0.403	0.219	0.527		

Pearson r value: >0.5 | >0.6 | >0.7 | >0.8
