# Supplementary material for: Correlations of Behavioral Deficits with Brain Pathology Assessed through Longitudinal MRI and Histopathology in the R6/1 Mouse Model of Huntington’s Disease
Source: PLoS One. 2013 Dec 19;8(12):e84726. doi: 10.1371/journal.pone.0084726 (PMC3868608; doi:10.1371/journal.pone.0084726)
Supplement: Table S4 — Correlations of behavioral measures taken between 15 and 19 weeks. Correlations of performance at behavioral tasks tested between 15 and 19 weeks, presented as Pearson r values. LMA = locomotor activity in an open field, GS FL = grip strength of the forelimbs, GS 4L = grip strength of the fore- and hind limbs, TM CL = swimming T-maze cue learning, TM CR = swimming T-maze cue reversal. *Statistically significant after Bonferroni Correction (adjusted p value 0.005). (PDF) [file pone.0084726.s005.pdf]

		LMA	GS FL	GS 4L	TM CL	TM CR	
WT males	LMA		-0.333	0.213	-0.125	-0.041	WT females
	GS FL	0.534		0.223	0.653	0.35	
	GS 4L	0.437	0.762		0.588	-0.306	
	TM CL	-0.08	0.056	0.016		-0.092	
	TM CR	0.28	-0.118	0.026	0.291		
R6/1 males	LMA		-0.447	0.461	-0.507	-0.799	R6/1 females
	GS FL	-0.27		0.108	-0.335	0.578	
	GS 4L	-0.733	0.556		-0.195	-0.541	
	TM CL	0.09	0.268	-0.122		0.173	
	TM CR	0.089	0.459	0.017	0.556		
WT & R6/1 males	LMA		-0.311	0.176	-0.229	-0.344	WT & R6/1 females
	GS FL	0.463		0.565	-0.388	-0.067	
	GS 4L	0.279	0.79*		-0.433	-0.669*	
	TM CL	-0.049	0.057	-0.093		0.472	
	TM CR	-0.212	-0.279	-0.383	0.542		

Pearson r value >0.5 >0.6 >0.7 >0.8
