# Supplementary material for: Correlations of Behavioral Deficits with Brain Pathology Assessed through Longitudinal MRI and Histopathology in the R6/1 Mouse Model of Huntington’s Disease
Source: PLoS One. 2013 Dec 19;8(12):e84726. doi: 10.1371/journal.pone.0084726 (PMC3868608; doi:10.1371/journal.pone.0084726)
Supplement: Table S5 — Correlations of MRI measures. Correlations of measures of pathological burden assessed through MRI across time, presented as Pearson r values. STR = striatum, CTX = cortex, HIPP = hippocampus, CC = corpus callosum, WB = whole brain, MUSC = muscle tissue. *Statistically significant after Bonferroni Correction (adjusted p value 0.0011). (PDF) [file pone.0084726.s006.pdf]

Group (males)	Measure	Region	Volumetry STR	Volumetry CTX	Volumetry HIPP	Volumetry CC	Volumetry WB	T2 relaxivity STR	T2 relaxivity CTX	T2 relaxivity HIPP	T2 relaxivity CC	T2 relaxivity MUSC	Group (females)
WT males	Volumetry	STR		0.419	0.102	0.221	0.316	0.355	0.414	0.528	0.198	0.143	WT females
		CTX	0.382		0.137	0.412	0.47	-0.164	0.045	0.242	0.003	0.252	
		HIPP	-0.056	0.037		0.355	0.44	0.01	0.334	0.282	-0.446	-0.165	
		CC	-0.063	-0.212	0.033		0.529	0.136	0.27	0.165	0.304	-0.032	
		WB	0.218	0.588	0.366	-0.157		-0.087	0.2	0.26	-0.074	0.405	
	T2 relaxivity	STR	-0.04	-0.209	0.36	0.115	-0.172		0.85*	0.666	0.414	0.203	
		CTX	-0.047	-0.291	0.199	0.071	-0.379	0.862*		0.887*	0.08	0.019	
		HIPP	0	-0.179	-0.044	0.116	-0.288	0.595	0.782*		-0.003	0.059	
		CC	0.08	-0.312	-0.114	0.028	-0.447	0.463	0.372	0.469		0.298	
		MUSC	-0.073	-0.092	0.013	-0.317	0	0.305	0.386	0.204	0.014		
R6/1 males	Volumetry	STR		0.501	0.481	0.124	0.352	0.177	0.195	0.126	0.146	-0.21	R6/1 females
		CTX	0.371		0.549	0.455	0.708*	-0.236	-0.253	-0.357	-0.001	-0.108	
		HIPP	0.14	0.475		0.354	0.693*	0.04	0.006	0.058	-0.356	0.186	
		CC	-0.002	-0.417	0.041		0.272	-0.291	-0.396	-0.266	-0.1	-0.004	
		WB	0.432	0.67	0.204	-0.049		-0.085	-0.027	-0.223	-0.353	0.215	
	T2 relaxivity	STR	-0.273	-0.386	-0.19	0.09	-0.508		0.868*	0.907*	0.072	-0.241	
		CTX	-0.257	-0.533	-0.178	0.005	-0.756*	0.849*		0.854*	-0.017	-0.022	
		HIPP	0.07	-0.378	-0.122	0.061	-0.536	0.854*	0.842*		0.056	-0.111	
		CC	-0.483	-0.456	-0.239	0.286	-0.111	0.009	0.089	-0.216		-0.16	
		MUSC	-0.129	-0.478	-0.635	0.133	0.021	0.023	0.075	-0.075	0.303		
WT & R6/1 males	Volumetry	STR		0.564*	0.438	0.155	0.487	0.333	0.276	0.167	0.226	0.028	WT & R6/1 females
		CTX	0.607*		0.599*	0.302	0.757*	-0.013	-0.085	-0.289	0.097	0.088	
		HIPP	0.435	0.513*		0.231	0.754*	0.163	0.143	-0.072	-0.21	0.025	
		CC	0.209	-0.023	0.263		0.272	-0.037	-0.029	-0.021	0.2	-0.025	
		WB	0.637*	0.768*	0.64*	0.199		0.084	0.081	-0.207	-0.024	0.267	
	T2 relaxivity	STR	0.207	0.069	0.401	0.262	0.175		0.846*	0.682*	0.3	0.012	
		CTX	0.029	-0.203	0.17	0.119	-0.186	0.837*		0.811*	0.043	-0.001	
		HIPP	0.113	-0.167	0.024	0.122	-0.175	0.706*	0.82*		-0.026	-0.047	
		CC	0.106	-0.091	0.099	0.218	0.025	0.381	0.29	0.162		0.164	
		MUSC	-0.143	-0.311	-0.279	-0.107	-0.068	0.07	0.158	0.004	0.096		

Pearson r value: >0.5 | >0.6 | >0.7 | >0.8
