# Supplementary material for: Correlations of Behavioral Deficits with Brain Pathology Assessed through Longitudinal MRI and Histopathology in the R6/1 Mouse Model of Huntington’s Disease
Source: PLoS One. 2013 Dec 19;8(12):e84726. doi: 10.1371/journal.pone.0084726 (PMC3868608; doi:10.1371/journal.pone.0084726)
Supplement: Table S6 — Correlations of behavioral abnormalities against brain abnormalities determined through MRI. Correlations of behavioral data against age-matched MRI measures taken between either 6 and 12 weeks (matched to 9 week MRI data) and 15 and 19 weeks (matched to 17 week MRI data), presented as Pearson r values. RR = rotarod, LMA = locomotor activity in an open field, GS FL = grip strength of the forelimbs, GS 4L = grip strength of the fore- and hind limbs, TM CL = swimming T-maze cue learning, TM CR = swimming T-maze cue reversal, FC CS = fear conditioning cue recall (total immobility over 25 cue exposures), FC CT = fear conditioning contextual recall, OD = odor discrimination, SI = social interaction, STR = striatum, CTX = cortex, HIPP = hippocampus, CC = corpus callosum, WB = whole brain, MUSC = muscle tissue. *Statistically significant after Bonferroni Correction (adjusted p value 0.001). (PDF) [file pone.0084726.s007.pdf]

			Males										Females									
			Volumetry					T2 relaxometry					Volumetry					T2 relaxometry				
			STR	CTX	HIPP	CC	WB	STR	CTX	HIPP	CC	MUSC	STR	CTX	HIPP	CC	WB	STR	CTX	HIPP	CC	MUSC
Behaviour and MRI taken from 6 - 12 weeks	WTs	RR	0.043	0.181	0.279	0.123	-0.407	0.195	0.343	0.248	0.433	-0.305	0.313	0.556	0.264	0.246	0.453	-0.362	-0.022	-0.11	-0.245	0.067
		LMA	0.292	0.231	0.047	-0.113	0.016	0.206	0.512	0.705	0.354	-0.206	0.323	0.271	0.14	-0.043	0.499	-0.046	0.093	0.408	-0.092	0.482
		GS FL	-0.148	-0.457	0.18	-0.031	-0.174	0.223	0.359	0.423	0.439	0.289	-0.085	-0.059	-0.493	0.414	-0.101	-0.179	0.37	-0.323	0.194	-0.113
		GS 4L	-0.034	0.257	0.348	-0.468	0.318	-0.255	-0.176	0.124	0.052	0.226	0.141	0.023	-0.509	-0.037	0.167	-0.372	0.129	-0.41	0.026	-0.106
		TM CL	-0.002	-0.597	-0.377	-0.029	-0.05	-0.276	-0.406	-0.35	0.219	0.114	-0.406	-0.211	0.652	-0.353	-0.27	0.007	-0.507	-0.152	-0.53	-0.417
		TM CR	0.037	0.243	0.048	0.023	-0.065	-0.206	0.15	-0.038	-0.565	-0.401	-0.378	-0.128	0.099	0.067	-0.725	0.137	-0.271	-0.537	0.009	-0.842
		FC CS	0.063	0.312	0.247	-0.198	0.445	0.08	-0.146	0.006	0.241	-0.087	0.459	-0.043	-0.382	-0.288	-0.164	0.041	-0.349	-0.257	0.088	-0.189
		FC CT	0.211	0.489	0.478	0.105	0.007	0.011	-0.269	-0.525	0.061	-0.636	0.329	-0.54	-0.614	-0.426	-0.073	0.05	-0.512	-0.052	0.337	0.221
		OD	0.275	-0.147	0.19	-0.417	-0.253	0.072	-0.091	-0.083	0.634	-0.232	0.396	-0.38	-0.774	-0.202	-0.562	0.683	-0.06	0.197	0.857	0.102
		SI	0.175	-0.153	-0.041	-0.609	0.35	0.123	-0.162	0.107	0.289	0.349	-0.626	-0.374	0.404	-0.145	-0.14	0.126	-0.279	0.109	-0.266	-0.073
	R6/1s	RR	-0.559	0.115	0.174	-0.138	-0.03	0.344	0.238	0.281	0.382	-0.126	-0.56	-0.365	0.105	0.076	-0.183	0.17	-0.037	0.198	0.166	0.472
		LMA	-0.409	-0.689	-0.415	-0.08	-0.497	0.699	0.575	0.517	-0.377	0.201	0.375	-0.076	-0.313	-0.001	-0.22	0.127	0.243	0.101	0.168	-0.361
		GS FL	-0.332	-0.268	-0.848	-0.511	-0.632	0.338	0.533	-0.038	0.371	0.211	0.695	0.602	0.524	-0.095	0.818	0.054	0.279	0.018	-0.038	0.075
		GS 4L	-0.141	-0.296	-0.7	-0.706	-0.225	0.069	0.377	0.033	0.052	0.493	0.582	0.626	0.582	0.105	0.762	0.142	0.288	0.11	-0.039	0.309
		TM CL	-0.198	-0.183	-0.131	0.08	-0.14	0.58	0.294	0.612	-0.535	-0.256	0.474	0.088	-0.012	-0.019	-0.201	0.189	0.288	0.064	0.126	-0.305
		TM CR	-0.017	-0.408	-0.131	-0.161	0.148	-0.19	0.082	-0.134	-0.234	0.519	-0.043	0.014	0.213	0.295	-0.102	0.513	0.606	0.67	-0.21	-0.403
		FC CS	0.299	0.695	0.345	-0.324	0.036	-0.164	0.143	0.207	0.253	-0.551	-0.158	0.096	-0.297	-0.297	0.203	-0.827	-0.827	-0.838	0.232	0.455
		FC CT	0.317	-0.227	0.364	-0.028	-0.055	0.076	0.133	0.471	-0.493	0.061	-0.548	-0.262	-0.369	-0.208	0.049	-0.59	-0.714	-0.534	0.174	0.526
		OD	-0.178	-0.426	0.334	0.417	0.05	0.016	0.068	-0.123	-0.168	0.196	0.377	0.401	-0.216	-0.304	0.048	-0.55	-0.398	-0.616	-0.067	0.064
		SI	0.173	-0.277	0.301	0.627	0.171	-0.269	-0.277	-0.379	-0.103	0.136	-0.13	-0.512	-0.198	0.035	-0.363	0.2	0.223	0.3	0.515	-0.508
	WTs & R6/1s	RR	0.117	0.343	0.389	0.225	0.057	0.432	0.467	0.422	0.548	-0.204	0.173	0.502	0.449	-0.219	0.489	0.018	-0.088	-0.275	-0.08	0.375
		LMA	0.22	0.143	0.104	0.121	0.197	0.579	0.646	**0.702***	0.341	-0.01	0.375	0.3	0.148	-0.176	0.364	0.084	0.092	0.091	-0.021	0.325
		GS FL	-0.211	-0.322	-0.247	-0.223	-0.256	0.191	0.324	0.137	0.303	0.234	0.284	0.293	0.071	0.147	0.386	-0.017	0.25	-0.103	0.09	-0.008
		GS 4L	-0.223	-0.125	-0.213	-0.597	-0.079	-0.214	-0.079	-0.081	-0.103	0.395	0.344	0.445	0.158	-0.171	0.548	-0.014	0.134	-0.255	0.018	0.195
		TM CL	-0.38	-0.557	-0.418	-0.209	-0.367	-0.173	-0.331	-0.168	-0.331	-0.093	0.142	0.074	0.23	-0.183	-0.113	0.164	0.22	-0.044	-0.13	-0.241
		TM CR	0.108	0.087	0.056	0.038	0.106	-0.036	0.193	0.032	-0.273	0.108	-0.141	0.148	0.294	-0.034	-0.124	0.397	0.367	0.052	-0.059	-0.407
		FC CS	0.407	0.59	0.435	0.009	0.499	0.27	0.265	0.332	0.437	-0.342	0.067	0.264	-0.014	-0.334	0.285	-0.588	-0.795	**-0.761***	0.12	0.406
		FC CT	0.412	0.372	0.53	0.216	0.237	0.274	0.156	0.147	0.135	-0.243	0.185	-0.064	-0.168	-0.523	0.244	-0.143	-0.448	-0.37	0.282	0.501
		OD	-0.025	-0.255	0.183	-0.089	-0.152	-0.013	-0.055	-0.112	0.273	0.033	0.361	-0.038	-0.51	-0.139	-0.295	-0.122	-0.321	-0.246	0.471	-0.02
		SI	0.402	0.1	0.312	0.268	0.452	0.204	0.122	0.127	0.319	0.156	-0.467	-0.404	0.102	-0.036	-0.242	0.135	0.123	0.208	-0.051	-0.246
Behaviour and MRI taken from 15-19 weeks	WTs	LMA	0.262	0.52	0.526	-0.309	0.408	0.03	-0.051	-0.303	-0.163	-0.005	-0.42	-0.103	-0.228	0.328	0.415	-0.035	-0.008	-0.101	0.33	0.211
		GS FL	0.311	0.302	0.631	-0.393	-0.007	0.62	0.622	0.292	0.247	0.542	0.578	0.506	0.708	0.129	0.529	-0.457	-0.282	-0.213	-0.108	-0.053
		GS 4L	0.558	0.535	0.481	-0.573	0.365	0.275	0.267	0.044	0.083	0.599	0.09	0.346	0.404	0.074	0.503	-0.288	0.085	0.183	-0.464	-0.067
		TM CL	-0.199	-0.092	-0.195	-0.591	-0.433	-0.291	-0.047	-0.121	-0.381	0.275	0.542	0.531	0.39	0.086	0.659	-0.272	0.01	0.055	-0.18	0.129
		TM CR	-0.573	0.003	0.264	-0.377	0.303	-0.316	-0.266	-0.469	-0.598	0.098	0.54	0.541	0.417	0.464	0.104	-0.451	-0.185	-0.087	0.095	-0.389
	R6/1s	LMA	0.264	0.108	-0.164	0.636	0.325	0.554	0.255	0.431	-0.109	0.365	0.309	0.15	0.781	0.622	0.101	-0.766	-0.796	-0.432	0.049	0.416
		GS FL	-0.42	-0.471	-0.077	-0.214	0.29	-0.436	-0.525	-0.489	0.436	0.169	-0.061	-0.152	-0.282	0.125	0.272	0.587	0.38	0.199	-0.087	0.136
		GS 4L	-0.674	-0.398	0.093	-0.618	-0.408	-0.435	-0.191	-0.389	0.174	-0.091	-0.171	0.362	0.357	0.484	0.564	-0.458	-0.784	-0.612	-0.555	0.036
		TM CL	0.479	-0.387	-0.068	0.127	0.488	0.136	-0.21	0.263	0.093	-0.224	-0.405	0.237	-0.516	-0.057	-0.164	-0.058	0.114	0.043	-0.108	-0.394
		TM CR	-0.114	-0.728	-0.312	0.335	0.208	-0.439	-0.669	-0.446	0.475	0.004	0.272	0.073	-0.387	-0.272	-0.019	0.747	0.795	0.62	0.307	-0.243
	WT & R6/1s	LMA	0.464	0.562	0.503	0.144	0.553	0.35	0.047	-0.046	-0.095	0.071	-0.097	-0.014	0.145	0.333	0.145	-0.303	-0.384	-0.224	0.232	0.284
		GS FL	0.401	0.49	0.64	-0.116	0.481	0.391	0.144	-0.138	0.292	0.257	0.545	0.561	0.566	0.335	0.675	0.173	0.055	-0.224	0.058	-0.127
		GS 4L	0.436	0.593	0.637	-0.288	0.555	0.199	0.031	-0.246	0.138	0.163	0.487	0.729	0.776	0.408	0.816	0.013	-0.2	-0.387	-0.101	-0.212
		TM CL	0.025	-0.194	-0.159	-0.28	-0.131	-0.11	-0.126	0.118	-0.2	0.032	-0.448	-0.354	-0.661	-0.221	-0.5	-0.326	-0.001	0.208	-0.261	0.056
		TM CR	-0.607	-0.687	-0.556	-0.315	-0.491	-0.497	-0.323	-0.18	-0.182	0.097	-0.172	-0.321	-0.563	-0.117	-0.48	0	0.324	0.445	-0.051	-0.088

Pearson r value >0.5 >0.6 >0.7 >0.8
