# Supplementary material for: Correlations of Behavioral Deficits with Brain Pathology Assessed through Longitudinal MRI and Histopathology in the R6/1 Mouse Model of Huntington’s Disease
Source: PLoS One. 2013 Dec 19;8(12):e84726. doi: 10.1371/journal.pone.0084726 (PMC3868608; doi:10.1371/journal.pone.0084726)
Supplement: Table S7 — Correlations of mHTT abundance across different brain regions. Correlations of total detectable mHTT (Total aggregated mHTT) and nuclear inclusions (Nuc mHTT) across the six brain regions investigated in 19 week old R6/1 mice only, presented as Pearson r values. STR = striatum, CTX = cortex, DG = dentate gyrus, CA1 = hippocampal CA1 subfield, CA2 = hippocampal CA2 subfield, CA3 = hippocampal CA3 subfield. *Statistically significant after Bonferroni Correction (adjusted p value 0.0008). (PDF) [file pone.0084726.s008.pdf]

			Total mHTT						Nuc mHTT						
			STR	CTX	DG	CA1	CA2	CA3	STR	CTX	DG	CA1	CA2	CA3	
**R6/1 males**	**Total mHTT**	**STR**		*0.552*	*0.73*	*0.641*	*0.644*	*0.712*	*0.617*	*-0.392*	*0.602*	*0.561*	*0.649*	*0.595*	***R6/1 females***
		**CTX**	0.769		*0.193*	*0.233*	*0.008*	*0.526*	*-0.268*	*0.228*	*0.215*	*0.516*	*0.626*	*-0.06*	
		**DG**	0.801	0.674		*0.805*	*0.83*	***0.957****	*0.625*	*0.054*	*0.405*	*0.665*	*0.95*	*0.795*	
		**CA1**	0.791	0.439	0.781		***0.912****	*0.88*	*0.523*	*-0.231*	*0.503*	*0.882*	*0.745*	*0.824*	
		**CA2**	0.188	0.111	0.654	0.447		*0.826*	*0.771*	*-0.111*	*0.691*	***0.94****	*0.946*	***0.976****	
		**CA3**	0.452	0.115	0.6	0.851	0.741		*0.452*	*0.01*	*0.397*	*0.793*	*0.897*	*0.766*	
	**Nuc mHTT**	**STR**	0.537	0.602	0.43	0.474	0.036	0.073		*-0.114*	*0.814*	*0.471*	*0.163*	*0.858*	
		**CTX**	0.387	0.675	0.154	0.176	-0.188	-0.188	0.85		*-0.005*	*0.051*	*-0.05*	*-0.004*	
		**DG**	0.459	0.353	0.359	0.547	0.064	0.265	**0.911***	0.652		*0.765*	*0.062*	*0.737*	
		**CA1**	0.193	0.23	0.57	0.576	0.725	0.562	0.876	0.32	0.861		*0.664*	*0.865*	
		**CA2**	-0.274	-0.341	-0.085	0.278	0.465	0.491	0.029	-0.001	0.13	0.489		*0.881*	
		**CA3**	0.156	-0.032	0.19	0.601	0.303	0.621	0.54	0.323	0.741	0.746	0.712		
**R6/1 males & females**	**Total mHTT**	**STR**	-	0.571	0.652	0.673	0.52	0.651	0.593	-0.066	0.462	0.443	0.31	0.503	
		**CTX**	0.571	-	0.521	0.291	-0.112	0.313	0.103	0.534	0.323	0.354	0.088	-0.108	
		**DG**	0.652	0.521	-	**0.753***	0.445	0.658	0.45	0.116	0.361	0.559	0.215	0.403	
		**CA1**	0.673	0.291	**0.753***	-	**0.753***	**0.852***	0.504	-0.028	0.511	0.749	0.496	0.716	
		**CA2**	0.52	-0.112	0.445	**0.753***	-	**0.784***	0.603	-0.135	0.273	**0.822***	**0.801***	**0.941***	
		**CA3**	0.651	0.313	0.658	**0.852***	**0.784***	-	0.355	-0.065	0.288	0.688	0.732	**0.722***	
	**Nuc mHTT**	**STR**	0.593	0.103	0.45	0.504	0.603	0.355	-	0.298	**0.825***	0.566	0.113	**0.764***	
		**CTX**	-0.066	0.534	0.116	-0.028	-0.135	-0.065	0.298	-	0.441	0.18	-0.02	0.056	
		**DG**	0.462	0.323	0.361	0.511	0.273	0.288	**0.825***	0.441	-	**0.777***	0.12	0.586	
		**CA1**	0.443	0.354	0.559	0.749	**0.822***	0.688	0.566	0.18	**0.777***	-	0.539	**0.804***	
		**CA2**	0.31	0.088	0.215	0.496	**0.801***	0.732	0.113	-0.02	0.12	0.539	-	**0.804***	
		**CA3**	0.503	-0.108	0.403	0.716	**0.941***	**0.722***	**0.764***	0.056	0.586	**0.804***	**0.804***	-	

Pearson r value: >0.5 | >0.6 | >0.7 | >0.8
