# Supplementary material for: Correlations of Behavioral Deficits with Brain Pathology Assessed through Longitudinal MRI and Histopathology in the R6/1 Mouse Model of Huntington’s Disease
Source: PLoS One. 2013 Dec 19;8(12):e84726. doi: 10.1371/journal.pone.0084726 (PMC3868608; doi:10.1371/journal.pone.0084726)
Supplement: Table S8 — Correlations of neuronal characteristics. Correlations of neuronal number (Neur no.), density (Neur dens.) and regional volume determined through stereological analyses of NeuN-strained brain sections in 19 week old mice, presented as Pearson r values. STR = striatum, M1 CTX = M1 cortex. *Statistically significant after Bonferroni Correction (adjusted p value 0.0033). (PDF) [file pone.0084726.s009.pdf]

Lower triangle (black)	Region	Measure	STR Neur no.	STR Neur dens.	STR Volume	M1 CTX Neur no.	M1 CTX Neur dens.	M1 CTX Volume	Upper triangle (blue italic)
WT males	STR	Neur no.		***0.876****	*0.068*	*0.035*	*0.15*	*-0.079*	*WT females*
WT males	STR	Neur dens.	**0.949***		*-0.42*	*0.291*	*0.52*	*-0.047*	*WT females*
WT males	STR	Volume	0.653	0.383		*-0.558*	*-0.787*	*-0.089*	*WT females*
WT males	M1 CTX	Neur no.	0.259	0.23	0.217		*0.668*	*0.764*	*WT females*
WT males	M1 CTX	Neur dens.	0.009	0.081	-0.219	0.091		*0.032*	*WT females*
WT males	M1 CTX	Volume	0.143	0.078	0.272	0.736	-0.602		*WT females*
R6/1 males	STR	Neur no.		*0.825*	*-0.462*	*0.638*	*0.652*	*-0.392*	*R6/1 females*
R6/1 males	STR	Neur dens.	**0.927***		***-0.869****	*0.585*	***0.878****	*-0.666*	*R6/1 females*
R6/1 males	STR	Volume	0.72	0.411		*-0.407*	*-0.832*	*0.729*	*R6/1 females*
R6/1 males	M1 CTX	Neur no.	0.079	0.107	-0.011		*0.611*	*-0.116*	*R6/1 females*
R6/1 males	M1 CTX	Neur dens.	0.333	0.616	-0.288	0.592		*-0.838*	*R6/1 females*
R6/1 males	M1 CTX	Volume	-0.283	-0.557	0.298	0.501	-0.398		*R6/1 females*
WT & R6/1 males	STR	Neur no.		*0.508*	*0.339*	*0.503*	*0.295*	*0.168*	*WT & R6/1 females*
WT & R6/1 males	STR	Neur dens.	**0.716***		*-0.622*	*0.166*	***0.77****	*-0.518*	*WT & R6/1 females*
WT & R6/1 males	STR	Volume	**0.75***	0.084		*0.269*	*-0.533*	***0.686****	*WT & R6/1 females*
WT & R6/1 males	M1 CTX	Neur no.	0.442	0.15	0.498		*0.392*	*0.539*	*WT & R6/1 females*
WT & R6/1 males	M1 CTX	Neur dens.	-0.156	0.254	-0.452	-0.034		*-0.55*	*WT & R6/1 females*
WT & R6/1 males	M1 CTX	Volume	0.416	-0.053	0.651	**0.785***	-0.639		*WT & R6/1 females*

Pearson r value	>0.5	>0.6	>0.7	>0.8
