# Supplementary material for: Correlations of Behavioral Deficits with Brain Pathology Assessed through Longitudinal MRI and Histopathology in the R6/1 Mouse Model of Huntington’s Disease
Source: PLoS One. 2013 Dec 19;8(12):e84726. doi: 10.1371/journal.pone.0084726 (PMC3868608; doi:10.1371/journal.pone.0084726)
Supplement: Table S9 — Correlations of mHTT levels versus MRI measures of brain abnormalities. Correlations of both post-mortem (at 19 weeks) total aggregated mHTT levels and nuclear inclusions (Nuc mHTT) against measures of brain pathology through MRI at 17 weeks, presented as Pearson r values. STR = striatum, CTX = cortex, DG = dentate gyrus, CA1 = hippocampal CA1 subfield, CA2 = hippocampal CA2 subfield, CA3 = hippocampal CA3 subfield, HIPP = hippocampus, CC = corpus callosum, WB = whole brain, MUSC = muscle tissue. (PDF) [file pone.0084726.s010.pdf]

			Volumetry					T2 relaxivity				
			STR	CTX	HIPP	CC	WB	STR	CTX	HIPP	CC	MUSC
Male R6/1s	Total mHTT	STR	0.054	0.03	-0.176	-0.571	0.042	0.654	0.579	0.646	-0.276	0.365
		CTX	0.268	0.179	-0.259	-0.486	-0.129	0.767	0.809	0.788	-0.374	0.33
		DG	0.233	-0.169	-0.555	-0.579	0.059	0.264	0.2	0.452	-0.303	0.382
		CA1	-0.021	-0.458	-0.309	-0.507	0.146	0.327	0.156	0.338	0.062	0.296
		CA2	0.587	-0.163	-0.554	0.018	0.319	-0.23	-0.465	0.116	-0.434	0.043
		CA3	0.189	-0.572	-0.333	-0.253	0.457	0.015	-0.229	0.074	0.293	0.143
	Nuc mHTT	STR	-0.186	-0.022	-0.19	-0.36	-0.332	0.422	0.479	0.296	-0.096	0.352
		CTX	0.058	0.209	-0.006	-0.091	-0.291	0.692	0.715	0.543	-0.207	0.196
		DG	-0.24	-0.093	-0.106	-0.311	-0.128	0.182	0.2	0.004	0.152	0.292
		CA1	0.218	-0.375	-0.537	-0.203	0.075	-0.334	-0.422	-0.145	0.129	0.199
		CA2	0.129	-0.615	-0.191	0.356	0.153	-0.149	-0.419	-0.105	0.279	-0.129
		CA3	-0.055	-0.501	-0.211	0.053	0.223	-0.014	-0.211	-0.151	0.45	0.179
Female R6/1s	*Total mHTT*	*STR*	*-0.634*	*0.125*	*-0.26*	*0.156*	*-0.169*	*-0.622*	*-0.65*	*-0.563*	*-0.217*	*-0.003*
		CTX	*-0.516*	*-0.198*	*-0.265*	*-0.076*	*0.128*	*0.243*	*0.073*	*0.221*	*0.053*	*0.328*
		DG	*-0.632*	*0.024*	*-0.674*	*-0.035*	*-0.302*	*-0.284*	*-0.401*	*-0.434*	*-0.258*	*-0.385*
		CA1	*-0.228*	*0.004*	*-0.2*	*0.558*	*-0.415*	*-0.454*	*-0.649*	*-0.279*	*-0.058*	*-0.149*
		CA2	*-0.309*	*0.097*	*-0.219*	*0.561*	*-0.251*	*-0.599*	*-0.737*	*-0.572*	*-0.276*	*-0.202*
		CA3	*-0.528*	*-0.074*	*-0.464*	*0.319*	*-0.268*	*-0.28*	*-0.451*	*-0.27*	*-0.08*	*-0.016*
	Nuc mHTT	*STR*	*-0.209*	*0.543*	*0.034*	*0.466*	*0.172*	*-0.727*	*-0.716*	*-0.809*	*-0.352*	*-0.132*
		CTX	*-0.103*	*0.024*	*-0.179*	*-0.071*	*0.571*	*0.533*	*0.366*	*0.165*	*-0.467*	*-0.179*
		DG	*-0.012*	*0.588*	*0.332*	*0.223*	*0.335*	*-0.455*	*-0.804*	*-0.538*	*-0.359*	*-0.269*
		CA1	*-0.096*	*0.172*	*0.056*	*0.303*	*-0.021*	*-0.195*	*-0.687*	*-0.229*	*-0.118*	*-0.155*
		CA2	*-0.63*	*-0.189*	*-0.895*	*-0.375*	*-0.528*	*0.169*	*0.147*	*0.178*	*0.467*	*-0.171*
		CA3	*-0.29*	*0.232*	*-0.202*	*0.567*	*-0.099*	*-0.59*	*-0.69*	*-0.628*	*-0.313*	*-0.189*

Pearson r value | >0.5 | >0.6 | >0.7 | >0.8
