# Supplementary material for: Correlations of Behavioral Deficits with Brain Pathology Assessed through Longitudinal MRI and Histopathology in the R6/1 Mouse Model of Huntington’s Disease
Source: PLoS One. 2013 Dec 19;8(12):e84726. doi: 10.1371/journal.pone.0084726 (PMC3868608; doi:10.1371/journal.pone.0084726)
Supplement: Table S10 — Correlations of neuronal characteristics versus MRI measures of brain abnormalities. Correlations of post-mortem neuronal number (Neur no.), density (Neur dens.) and regional volume determined through stereology on NeuN-stained brain sections against measures of brain pathology through MRI at 17 weeks of age, presented as Pearson r values. STR = striatum, M1 CTX = primary motor cortex, CTX = cortex, HIPP = hippocampus, CC = corpus callosum, WB = whole brain, MUSC = muscle tissue. *Statistically significant after Bonferroni Correction (adjusted p value 0.0033). (PDF) [file pone.0084726.s011.pdf]

Genotype	Sex	Region	Measure	Volumetry STR	Volumetry CTX	Volumetry HIPP	Volumetry CC	Volumetry WB	T2 relaxivity STR	T2 relaxivity CTX	T2 relaxivity HIPP	T2 relaxivity CC	T2 relaxivity MUSC
WT	Males	STR	Neur no.	-0.05	0.129	0.246	-0.208	-0.043	-0.356	-0.364	-0.382	-0.574	-0.252
WT	Males	STR	Neur dens.	-0.036	0.163	0.147	-0.206	-0.004	-0.389	-0.435	-0.475	-0.512	-0.225
WT	Males	STR	Volume	0.008	0.077	0.342	-0.109	-0.049	-0.17	-0.071	-0.036	-0.519	-0.239
WT	Males	M1 CTX	Neur no.	-0.184	0.002	0.34	0.41	0.566	-0.432	-0.588	-0.274	-0.37	-0.686
WT	Males	M1 CTX	Neur dens.	-0.874*	-0.675	0.083	0.216	-0.233	0.199	-0.061	-0.228	0.157	-0.149
WT	Males	M1 CTX	Volume	0.423	0.442	0.237	0.173	0.62	-0.429	-0.385	-0.032	-0.356	-0.42
WT	*Females*	*STR*	*Neur no.*	*-0.082*	*-0.124*	*0.243*	*-0.319*	*-0.121*	*-0.23*	*-0.222*	*-0.133*	*-0.71*	*-0.26*
WT	*Females*	*STR*	*Neur dens.*	*-0.238*	*-0.172*	*0.223*	*-0.372*	*-0.012*	*-0.139*	*-0.079*	*0.019*	*-0.758*	*-0.221*
WT	*Females*	*STR*	*Volume*	*0.315*	*0.101*	*-0.054*	*0.161*	*-0.207*	*-0.087*	*-0.216*	*-0.273*	*0.248*	*0.017*
WT	*Females*	*M1 CTX*	*Neur no.*	*-0.085*	*-0.282*	*-0.135*	*-0.65*	*-0.234*	*0.207*	*0.209*	*0.539*	*-0.331*	*-0.099*
WT	*Females*	*M1 CTX*	*Neur dens.*	*-0.141*	*-0.079*	*-0.034*	*-0.491*	*-0.104*	*0.289*	*0.515*	*0.614*	*-0.57*	*-0.003*
WT	*Females*	*M1 CTX*	*Volume*	*0.021*	*-0.29*	*-0.115*	*-0.436*	*-0.192*	*-0.09*	*-0.185*	*0.019*	*0.203*	*-0.129*
R6/1	Males	STR	Neur no.	0.144	-0.156	0.137	0.287	0.564	0.489	0.164	0.246	0.36	0.134
R6/1	Males	STR	Neur dens.	-0.077	-0.115	0.219	0.347	0.53	0.257	-0.025	-0.031	0.435	0.146
R6/1	Males	STR	Volume	0.544	-0.128	-0.103	0.06	0.427	0.692	0.418	0.644	0.056	0.071
R6/1	Males	M1 CTX	Neur no.	-0.146	-0.096	0.404	-0.458	-0.327	-0.141	-0.227	0.084	-0.327	-0.636
R6/1	Males	M1 CTX	Neur dens.	-0.294	0.001	0.355	0.05	0.184	-0.586	-0.758	-0.534	0.154	-0.261
R6/1	Males	M1 CTX	Volume	0.155	-0.106	0.073	-0.604	-0.573	0.44	0.545	0.646	-0.526	-0.448
R6/1	*Females*	*STR*	*Neur no.*	*-0.367*	*-0.245*	*-0.42*	*-0.128*	*-0.369*	*-0.109*	*-0.434*	*-0.293*	*-0.312*	*-0.471*
R6/1	*Females*	*STR*	*Neur dens.*	*-0.197*	*-0.073*	*-0.504*	*0.068*	*-0.511*	*-0.168*	*-0.352*	*-0.254*	*-0.18*	*-0.616*
R6/1	*Females*	*STR*	*Volume*	*-0.083*	*-0.107*	*0.462*	*-0.06*	*0.554*	*0.021*	*0.069*	*0.005*	*-0.084*	*0.636*
R6/1	*Females*	*M1 CTX*	*Neur no.*	*-0.668*	*-0.122*	*-0.828*	*-0.309*	*-0.438*	*-0.002*	*-0.042*	*-0.096*	*0.184*	*-0.207*
R6/1	*Females*	*M1 CTX*	*Neur dens.*	*-0.086*	*-0.018*	*-0.494*	*0.15*	*-0.533*	*-0.122*	*-0.206*	*-0.145*	*0.208*	*-0.257*
R6/1	*Females*	*M1 CTX*	*Volume*	*-0.354*	*0.013*	*0.111*	*-0.231*	*0.392*	*0.026*	*0.089*	*0.039*	*-0.293*	*0.131*
WT & R6/1	Males	STR	Neur no.	0.423	0.485	0.562	0.126	0.546	0.215	-0.143	-0.176	-0.161	-0.137
WT & R6/1	Males	STR	Neur dens.	-0.111	-0.052	0.01	-0.006	0.003	-0.124	-0.235	-0.199	-0.191	-0.038
WT & R6/1	Males	STR	Volume	0.727*	0.752*	0.794*	0.219	0.803*	0.442	0.022	-0.059	-0.02	-0.146
WT & R6/1	Males	M1 CTX	Neur no.	0.242	0.397	0.572	0.269	0.59	-0.015	-0.387	-0.184	-0.25	-0.577
WT & R6/1	Males	M1 CTX	Neur dens.	-0.7*	-0.571	-0.241	0.062	-0.394	-0.236	-0.288	-0.258	0.094	-0.155
WT & R6/1	Males	M1 CTX	Volume	0.621	0.667*	0.603	0.157	0.709*	0.145	-0.111	0.007	-0.209	-0.326
WT & R6/1	*Females*	*STR*	*Neur no.*	*0.232*	*0.32*	*0.427*	*-0.025*	*0.329*	*0.036*	*-0.211*	*-0.3*	*-0.346*	*-0.395*
WT & R6/1	*Females*	*STR*	*Neur dens.*	*-0.318*	*-0.263*	*-0.344*	*-0.234*	*-0.389*	*-0.226*	*-0.272*	*-0.077*	*-0.488*	*-0.327*
WT & R6/1	*Females*	*STR*	*Volume*	*0.561*	*0.596*	*0.753**	*0.309*	*0.728**	*0.251*	*0.045*	*-0.267*	*0.273*	*0.008*
WT & R6/1	*Females*	*M1 CTX*	*Neur no.*	*0.214*	*0.362*	*0.328*	*-0.182*	*0.358*	*0.285*	*0.113*	*-0.017*	*0.044*	*-0.248*
WT & R6/1	*Females*	*M1 CTX*	*Neur dens.*	*-0.11*	*-0.068*	*-0.249*	*-0.119*	*-0.283*	*-0.022*	*-0.039*	*0.062*	*-0.136*	*-0.127*
WT & R6/1	*Females*	*M1 CTX*	*Volume*	*0.267*	*0.381*	*0.509*	*-0.029*	*0.559*	*0.182*	*0.048*	*-0.12*	*0.141*	*-0.126*

Pearson r value: >0.5 | >0.6 | >0.7 | >0.8
