# Supplementary material for: Correlations of Behavioral Deficits with Brain Pathology Assessed through Longitudinal MRI and Histopathology in the R6/1 Mouse Model of Huntington’s Disease
Source: PLoS One. 2013 Dec 19;8(12):e84726. doi: 10.1371/journal.pone.0084726 (PMC3868608; doi:10.1371/journal.pone.0084726)
Supplement: Table S11 — Correlations of mHTT levels versus behavioral performance. Correlations of post-mortem (19 weeks) total mHTT and nuclear inclusions (Nuc mHTT) against behavioral phenotypes exhibited by R6/1 mice between 15 and 19 weeks of age, presented as Pearson r values. LMA = locomotor activity in an open field, GS FL = grip strength of the forelimbs, GS 4L = grip strength of the fore- and hind limbs, TM CL = swimming T-maze cue learning, TM CR = swimming T-maze cue reversal, STR = striatum, CTX = cortex, DG = dentate gyrus, CA1 = hippocampal CA1 subfield, CA2 = hippocampal CA2 subfield, CA3 = hippocampal CA3 subfield. *Statistically significant after Bonferroni Correction (adjusted p value 0.0017). (PDF) [file pone.0084726.s012.pdf]

		Male R6/1s					Female R6/1s				
		LMA	GS FL	GS 4L	TM CL	TM CR	*LMA*	*GS FL*	*GS 4L*	*TM CL*	*TM CR*
Total mHTT	STR	0.16	0.179	-0.042	0.154	-0.327	*0.274*	*-0.367*	*0.511*	*0.396*	*-0.646*
	CTX	0.148	-0.431	-0.281	-0.051	-0.562	*-0.012*	*0.318*	*0.445*	*0.128*	*-0.225*
	DG	-0.107	0.2	0.055	0.24	-0.098	*-0.355*	*0.205*	*0.337*	*0.374*	*-0.07*
	CA1	0.014	0.494	0.092	0.519	0.308	*0.293*	*0.072*	*0.472*	*0.142*	*-0.303*
	CA2	-0.038	0.182	-0.181	0.709	0.47	*0.219*	*0.184*	*0.664*	*0.14*	*-0.286*
	CA3	-0.131	0.591	0.099	0.79	0.547	*0.007*	*0.321*	*0.476*	*0.163*	*-0.204*
Nuc mHTT	STR	0.23	-0.172	-0.301	-0.278	-0.013	*0.246*	*-0.145*	*0.677*	*0.269*	*-0.324*
	CTX	0.464	-0.593	-0.589	-0.245	-0.222	*-0.577*	*0.79*	*0.247*	*-0.05*	*0.551*
	DG	0.109	0.133	-0.234	-0.163	0.206	*0.442*	*-0.202*	*0.872*	*0.021*	*-0.516*
	CA1	-0.221	0.118	-0.16	0.244	0.514	*0.365*	*0.18*	*0.77*	*-0.209*	*-0.377*
	CA2	0.215	0.136	-0.199	0.653	**0.925***	*-0.484*	*0.14*	*-0.073*	*0.573*	*0.122*
	CA3	0.186	0.324	-0.271	0.398	0.781	*0.109*	*0.289*	*0.717*	*0.18*	*-0.195*

Pearson r value: >0.5 | >0.6 | >0.7 | >0.8
