# Supplementary material for: Correlations of Behavioral Deficits with Brain Pathology Assessed through Longitudinal MRI and Histopathology in the R6/1 Mouse Model of Huntington’s Disease
Source: PLoS One. 2013 Dec 19;8(12):e84726. doi: 10.1371/journal.pone.0084726 (PMC3868608; doi:10.1371/journal.pone.0084726)
Supplement: Table S12 — Correlations of neuronal characteristics versus behavioral performance. Correlations of post-mortem (19 weeks) neuronal number (Neur no.), density (Neur dens.) and regional volume determined through stereology on NeuN-stained brain sections against behavioral performance recorded between 15 and 19 weeks of age, presented as Pearson r values. LMA = locomotor activity in an open field, GS FL = grip strength of the forelimbs, GS 4L = grip strength of the fore- and hind limbs, TM CL = swimming T-maze cue learning, TM CR = swimming T-maze cue reversal, STR = striatum, M1 CTX = primary motor cortex. *Statistically significant after Bonferroni Correction (adjusted p value 0.0033). (PDF) [file pone.0084726.s013.pdf]

			Male					Female				
			LMA	GS FL	GS 4L	TM CL	TM CR	LMA	GS FL	GS 4L	TM CL	TM CR
WTs	STR	Neur no.	0.211	0.024	-0.15	0.762	0.115	-0.555	0.167	0.103	0.222	-0.274
		Neur dens.	0.284	-0.068	-0.243	0.71	0.071	-0.335	0.134	0.432	0.236	-0.51
		Volume	-0.015	0.209	0.147	0.5	0.151	-0.335	-0.015	-0.701	-0.074	0.483
	M1 CTX	Neur no.	0.191	-0.487	-0.291	-0.225	0.509	-0.16	0.262	0.092	0.022	-0.246
		Neur dens.	-0.252	-0.353	-0.709	-0.151	0.191	0.061	-0.107	0.499	-0.007	-0.42
		Volume	0.347	-0.125	0.28	-0.116	0.286	-0.267	0.48	-0.27	0.071	0.032
R6/1s	STR	Neur no.	0.569	0.234	-0.446	0.556	0.328	-0.085	0.243	0.368	-0.134	-0.175
		Neur dens.	0.536	0.424	-0.321	0.355	0.371	-0.354	0.267	0.069	0.149	0.126
		Volume	0.393	-0.212	-0.546	0.687	0.098	0.544	-0.163	0.37	-0.333	-0.432
	M1 CTX	Neur no.	-0.355	0.459	0.409	0.45	0.389	-0.405	0.143	0.048	0.415	0.035
		Neur dens.	-0.09	0.769	0.217	0.159	0.447	-0.277	0.436	-0.085	0.033	0.251
		Volume	-0.35	-0.304	0.23	0.329	-0.145	0.099	-0.494	0.25	0.337	-0.369
WTs & R6/1s	STR	Neur no.	0.524	0.412	0.208	0.446	-0.213	-0.329	0.468	0.529	-0.351	-0.468
		Neur dens.	0.282	0.028	-0.273	0.532	0.336	-0.333	0.071	0.043	0.298	0.101
		Volume	0.503	0.58	0.546	0.117	-0.593	0.028	0.442	0.518	**-0.652***	-0.541
	M1 CTX	Neur no.	0.31	0.118	0.258	-0.1	-0.141	-0.212	0.508	0.521	-0.304	-0.434
		Neur dens.	-0.369	-0.251	-0.511	0.06	0.473	-0.139	0.252	0.141	0.059	0.122
		Volume	0.48*	0.293	0.55	-0.128	-0.398	-0.071	0.272	0.418	-0.255	-0.516

Pearson r value: >0.5 | >0.6 | >0.7 | >0.8
